# Physiological and iTRAQ-based proteomic analyses reveal the function of exogenous γ-aminobutyric acid (GABA) in improving tea plant (*Camellia sinensis* L.) tolerance at cold temperature

**DOI:** 10.1186/s12870-019-1646-9

**Published:** 2019-01-30

**Authors:** Xujun Zhu, Jieren Liao, Xingli Xia, Fei Xiong, Yue Li, Jiazhi Shen, Bo Wen, Yuanchun Ma, Yuhua Wang, Wanping Fang

**Affiliations:** 1 College of Horticulture, Nanjing Agricultural University, No.1 Weigang, Nanjing, Jiangsu Province 210095 People’s Republic of China; 2 Wuxi NextCODE Genomics, 288 Fute Zhong Road, Shanghai, 200131 People’s Republic of China

**Keywords:** Differentially abundant proteins, Gene expression, GABA shunt, Metabolic pathway, Carbon and nitrogen metabolism

## Abstract

**Background:**

Internal γ-Aminobutyric Acid (GABA) interacting with stress response substances may be involved in the regulation of differentially abundant proteins (DAPs) associated with optimum temperature and cold stress in tea plants (*Camellia sinensis* (L.) O. Kuntze).

**Results:**

Tea plants supplied with or without 5.0 mM GABA were subjected to optimum or cold temperatures in this study. The increased GABA level induced by exogenous GABA altered levels of stress response substances – such as glutamate, polyamines and anthocyanins – in association with improved cold tolerance. Isobaric tags for relative and absolute quantification (iTRAQ) – based DAPs were found for protein metabolism and nucleotide metabolism, energy, amino acid transport and metabolism other biological processes, inorganic ion transport and metabolism, lipid metabolism, carbohydrate transport and metabolism, biosynthesis of secondary metabolites, antioxidant and stress defense.

**Conclusions:**

The iTRAQ analysis could explain the GABA-induced physiological effects associated with cold tolerance in tea plants. Analysis of functional protein–protein networks further showed that alteration of endogenous GABA and stress response substances induced interactions among photosynthesis, amino acid biosynthesis, and carbon and nitrogen metabolism, and the corresponding differences could contribute to improved cold tolerance of tea plants.

**Electronic supplementary material:**

The online version of this article (10.1186/s12870-019-1646-9) contains supplementary material, which is available to authorized users.

## Background

Tea is one of the most popular non-alcoholic beverages all over the world, and tea plants (*Camellia sinensis* (L.) O. Kuntze) are one of the most vital commercial crops in many countries such as China, India, Sri Lanka and Kenya [1]. As an evergreen woody plant, tea is grown from tropical to subtropical areas. Tea plants continuously suffer from a wide variety of environmental stresses, including low temperature, which affects growth, survival and geographical distribution [2]. Cold stress can easily disturb photosynthesis process which is the most important process required for plant production [3, 4]. Intensive studies have explored the molecular, cellular and physiological events that regulate the growth of tea plants and differentiation under cold stress [5–7]. For genetic engineering and plant breeding, it is vital to understand the mechanism for regulating tea plants’ growth under cold stress. Extensive molecular and genetic studies have greatly contributed to help us understand how plants survive and grow at low temperature. Various genes and metabolic pathways have been identified that function in enhancing cold tolerance. Well-known stress tolerance mechanisms including damage to membranes, GABA accumulation and its signaling pathways leading to osmotic adjustment, and other mechanisms associated with various secondary messengers, GABA also leads to transcriptional and post-transcriptional regulation [5, 7, 8]. Numerous genes encoding transcription factors and other metabolites related to cold stress have been reported and used for genetic engineering of plants with enhanced cold tolerance [9, 10]. Genome-wide analysis through transcriptomics and proteomics has been employed to illuminate events of some molecular types in environmental stress reaction, and the tea tree genome was sequenced very recently [11]. Two dimensional electrophoresis and matrix-assisted laser desorption/ionization time-of-flight mass spectrometry have been used to analyze drought stress and ABA-responsive proteins in tea plants [12]. However, there is a lack of proteome analysis on tea plant response to low temperature.

GABA, a non-protein amino acid, is widely found in bacteria through yeasts to vertebrates, was detected in plants almost 60 years ago [13]. In plant cells, there are two ways of GABA compound have been concretely depicted: through the GABA shunt pathway involving α-decarboxylation of glutamate from the chemical reactions of glutamate decarboxylase (GAD); and the degradation reaction of polyamines (PAs) from the chemical reactions of diamine oxidase (DAO) and polyamine oxidase (PAO). GABA is converted to another kind of molecule, succinic semialdehyde, through GABA transaminase (GABA-T) secondly, the oxidizing reaction of succinic semialdehyde to succinate and then entering the tricarboxylic acid (TCA) cycle. Thus alleviating mitochondrial electron transport chain for sufficient ATP production, and minimizing accumulation of hydrogen peroxide (H_2_O_2_) under abiotic stress [14]. As a signal molecule, GABA also takes greatly effect in adjusting stress reactions [15, 16]. Song et al. [17] reported that GABA could led the alleviation of oxidative damage induced by proton and aluminum stresses on barley seedlings. Expression of genes involved in H_2_O_2_ generation could be regulated by GABA in *Caragana intermedia* roots under saline conditions [18]. When tea plants experience low-temperature conditions, various mechanisms are invoked, including antioxidant capabilities, osmotic adjustment, photosynthetic rate reduction, and GABA accumulation [19]. These processes involve expression of cold-responsive genes, many of which are regulated by GABA. However, it is largely unknown which proteins are regulated by GABA for tea plants subject to optimum and low temperatures.

In the present study, we investigated the tolerance mechanisms by GABA through physiological and iTRAQ-based proteomic analyses under optimum and low temperatures in tea plants. The results should guide agricultural planning and minimize tea yield reductions due to low temperatures in early spring. Our study also suggests that GABA could play a role in carbon and nitrogen metabolism under cold stress.

## Methods

### Chemicals

GABA, putrescine, spermidine, spermine, glutamate and standard amino acids were bought from ShangHai YuanYe Bio-technology co.Ltd. (Shanghai, China). All other reagents were of analytical grade.

### Plant materials and treatments

One-year-old tea plants (*C. sinensis* cv. Longjingchangye) were cultivated in Nanjing Agricultural University. During the experiment, tea plants were well planted in a growth room with a photoperiod regimen composed of 14 h of light (25 °C, 200 μmol m^− 2^ s^− 1^, 75% humidity) and 10 h of dark (25 °C, 75% humidity). These tea plants had been grown up for 3 weeks in the nutritive medium introduced by Wan et al. [20] and after that, the plants were moved to a exposure to the four different ways of handling as explained below.

In preliminary experiments, the concentrations of GABA were 1.0, 3.0 and 5.0 mM. Then, 5.0 mM GABA was selected for this experiment according to free proline content. In the treatment (T1), tea plants were treated at 25 °C; in the treatment (T2), GABA was applicated into nutrient solution at 25 °C; in the treatment (T3), tea plants were treated at 4 °C; in the treatment (T4), GABA was applicated into nutrient solution at 4 °C. After 0, 4, 7 days, one bud and two or three leaves were picked and stored at − 80 °C. Physiological parameters were measured by 0, 4 and 7 days, and iTRAQ was measured by the day 7.

### Extraction and analysis of peroxidase (POD), superoxide dismutase (SOD), and catalase (CAT)

The techniques for extraction and analysis of POD, SOD were described by Wu et al. [21], and CAT analysis was described by Parviz Malekzadeh et al. [22].

### Determination of free proline and MDA content

The method for extraction and analysis of free proline and MDA content were described by Wang et al. [23].

### Determination of polyamines content

The method for extraction of free polyamines and analysis for HPLC were described by Liao et al. [14]. The HPLC column (device) employed for polyamines determination is ACQUITY UPLC HSS T3 (100 mm × 2.1 mm × 1.8 μm).

### Analysis of amino acid component contents

The method for extraction amino acid component were described by Zhao et al. [24], with slightly modified and analysis for amino acid analyzer.

### DAO and PAO activity assay

DAO and PAO enzymes were extracted and the activity was determined using the method described by Hu et al. [25], with slight modifications. Plant leaves (0.5 g) were ground with mortar and pestle at 4 °C in 1.6 mL 0.1 M sodium phosphate buffer (pH 6.5) containing 5% PVP (*w*/*v*). Homogenates were centrifuged at 10, 000×g for 20 min at 4 °C. Reaction mixtures (3.0 mL) contained 2.5 mL 0.1 M sodium phosphate buffer (pH 6.5), 0.1 mL crude enzyme extracts, 0.1 mL peroxidase (250 U·mL^− 1^), and 0.2 mL 4-aminoantipyrine/N, N-dimethylaniline. 0.1 mL 20 mM putrescine and spermidine was added for the reaction initiation for DAO and PAO activity, respectively. One activity unit of the enzyme was set as a 0.01 change of absorbance at 555 nm.

### GABA-T and GAD activity assay

The GABA-T and GAD enzymes were extracted and analyzed according to the method of Wang et al. [23] with slight modifications.

### Measurement of chlorophyll a fluorescence transients OJIP and JIP-test and SPAD value

One-year-old tea plants with four to five new shoots were adapted in the dark for 1 h. Chlorophyll a fluorescence transient responses were measured by an analysis instrument of plant effect (Handy PEA fluorometer, Hansatech Instruments Ltd., UK). In this study, the method to summarize the formulae and interpretative statement of the record of the OJIP curves, and the well selected JIP-test parameters was represented by Chen [26]. The SPAD values were managed with a SPAD meter ‘SPAD- 502’ (Konica Minolta, Japan) described by Wu et al. [27]. The average SPAD values was regarded as the relative chlorophyll content.

### Gene expression analysis

The method of RNA extraction, reverse transcription of cDNA, qRT-PCR analysis reactions and calculation of the relative expression levels were described by Liao et al. [14] The specific primer pairs used for qRT-PCR are listed in Additional file 1: Table S1. Let the *C. sinensis* β-actin gene as a reference as genetic type. All tests were made for three times in a same way with independent sample data.

### Protein extraction, digestion, and iTRAQ labeling

Use tea leaf samples for protein extraction. Firstly, the 0.1 g frozen plant tissue was ground and then placed in a 10 ml tube containing 1 mL phenol extraction buffer and mix, and the sample was held at 25 °C for 10 min. Add 1 mL phenol saturated with Tris-HCl (pH 8.0), and shake the mixture for 40 min at 4 °C. Centrifuge the sample at 15,000 *g* at 4 °C for 30 min, then dissolve the dry pellet in extraction buffer, thoroughly mixed by vortex and then placed in cold 0.1 M ammonium acetate - methanol solution for 12 h at − 20 °C. Then use five volumes of the collected phenolic phase, centrifuge at 12000 g for 10 min at 4 °C, collect the sediment, repeat third times, at last dry it at room temperature for 2 min. After adding resuspend the sediment in 300 μL lysate solution at RT for 3 h, centrifuge at 12000 g for 10 min at RT, collect the supernatant, repeat one more time. The supernatant was the extracted protein solution. Store the sample at − 70 °C. Measurement of protein were performed by BCA method, [28] and the method of measuring the quality of the protein samples was described by SDS-PAGE gel electrophoresis [29]. FASP method was used to digest protein [30]. For each sample, dissolve 100 μg of total protein in 120 μL reducing buffer (10 mM DTT, 8 M Urea, 100 mM TEAB, pH 8.0). The samples were labeled with iTRAQ Reagent-4 plex Multiplex Kit (AB Sciex) after being reduced, alkylated, and trypsin-digested. Four samples were labeled with iTRAQ tags 113 (T1), 114 (T2), 115 (T3), and 116 (T4), with three biological replicates.

### Mass spectrometry analysis

Use a Triple TOF 5600 mass spectrometer (SCIEX, USA) equipped with a Nanospray III source (SCIEX, USA) to perform all analyses. A capillary C_18_ trap column (3 cm × 100 μm) was used to loaded samples and then separate them by a C_18_ column (15 cm × 75 μm) on an Eksigent nanoLC-1D plus system (SCIEX, USA). The flow rate was 300 nL/min and linear gradient was 90 min (from 5 to 85% B over 67 min; mobile phase A = 2% ACN/0.1% FA and B = 95% ACN/0.1% FA).

Acquire data with a 2.4 kV ion spray voltage, 35 psi curtain gas, 5 psi nebulizer gas, and an interface heater temperature of 150 °C. The MS scanned between 400 and 1500 with an accumulation time of 250 ms. For IDA, 30 MS/MS spectra (80 ms each, mass range 100–1500) of MS peaks above intensity 260 and having a charge state of between 2 and 5 were acquired. Use a rolling collision energy voltage for CID fragmentation for MS/MS spectra acquisitions. Mass was dynamically excluded for 22 s.

### Protein identification and quantification

For iTRAQ data analysis, the Protein Pilot 5.0 Software was performed against amino acid sequences (the number of entries is 133,689), which were further translated by the unigene in tea plant transcriptomics database (https://submit.ncbi.nlm.nih.gov/subs/sra/SUB4781815/overview). iTRAQ proteins with unique peptides (FDR < 1%) were corrected for further analysis. The differentially abundant proteins (DAPs) were identified on the basis of the ratios of differently labeled proteins (T2/T1, treated with 5 mM GABA under optimum temperature; T3/T1, treated under low temperature compared to that under optimum temperature; T4/T1, treated with 5 mM GABA under low temperature compared to that under optimum temperature; T4/T2, treated with 5 mM GABA under low temperature compared to that with 5 mM GABA under optimum temperature and T4/T3, treated with 5 mM GABA under low temperature compared to that under low temperature). And the following DAP filter criteria was performed: fold change of > 1.5 or < 0.67 and *p* value < 0.05.

### Bioinformatics and annotations

For further functional analysis, search all the DAPs against UniProt *Arabidopsis thaliana* database. The best identity proteins were regarded as homologous (cut-off Evalue = 10^− 10^). Analyze gene ontology (GO) annotation with OmicsBean (http://www.omicsbean.cn/). The Kyoto Encyclopedia of Genes and Genomes (KEGG) database was employed to analyze the canonical biochemical pathways. Protein–protein interaction (PPI) networks was constructed on the basis of the STRING database [31] in order to better understanding on interaction functions of DAPs.

### Statistical analysis

The mean SE of the three independent experiments was considered as each value. All data were analyzed by SPASS 20.0(Windows, USA). Use Duncan’s test and ANOVA to determine significance. A probability level of 5% (*p* < 0.05) was regarded as statistically significant.

## Results

### Effect on endogenous GABA and glutamate contents

**Fig. 1:**
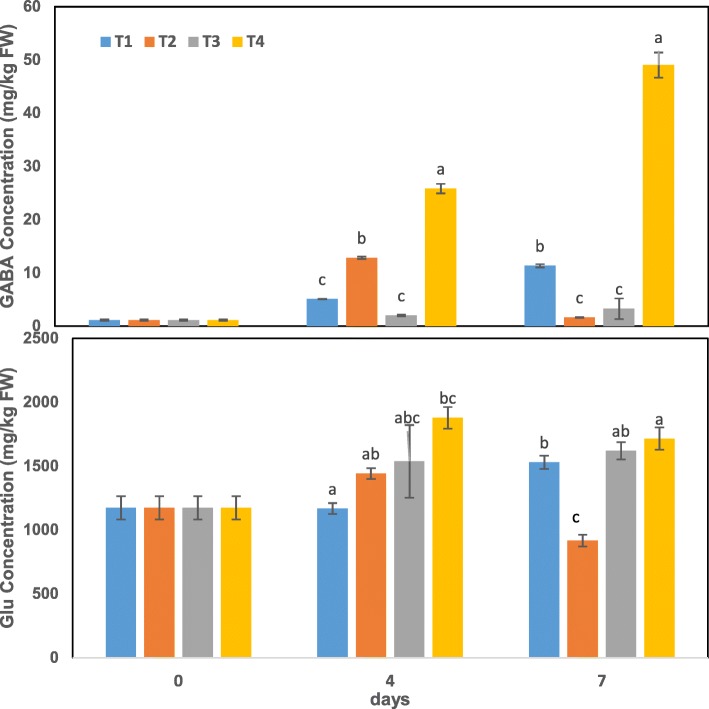
Changes in endogenous GABA and glutamate contents during treatments T1–T4 (25 °C, 25 °C + GABA, 4 °C and 4 °C + GABA for T1, T2, T3 and T4, respectively). Data represent the mean value ± standard deviation. Means with different letters significantly differ from each other (*p* ≤ 0.05). Glu, glutamate

The GABA and glutamate contents in tea leaves were determined at different temperatures in this study. Endogenous GABA content in tea leaves decreased significantly under low temperature at day 7 but showed no obvious change at day 4. However, glutamate content did not exhibit significantly change (Fig. 1).

In the presence of exogenous GABA, endogenous GABA content increased stably under cold stress, reaching its peak at 49.03 mg·kg^− 1^ FW at day 7 – a roughly 49-fold increment relative to the untreated control at 0 h. However, the precursor glutamate content increased sharply during days 0–4 and then decreased gradually. The GABA and glutamate contents showed almost the same trend at optimum temperature, both increasing during days 0–4 and then decreasing gradually (Fig. 1). Endogenous GABA content in tea plants changed significantly with application of exogenous GABA compared to without, for the two different temperatures; however, endogenous glutamate content varied significantly only at optimum temperature on day 7 (Fig. 1).

### SPAD value and analysis of tolerance by chlorophyll fluorescence transients and JIP-test

**Fig. 2:**
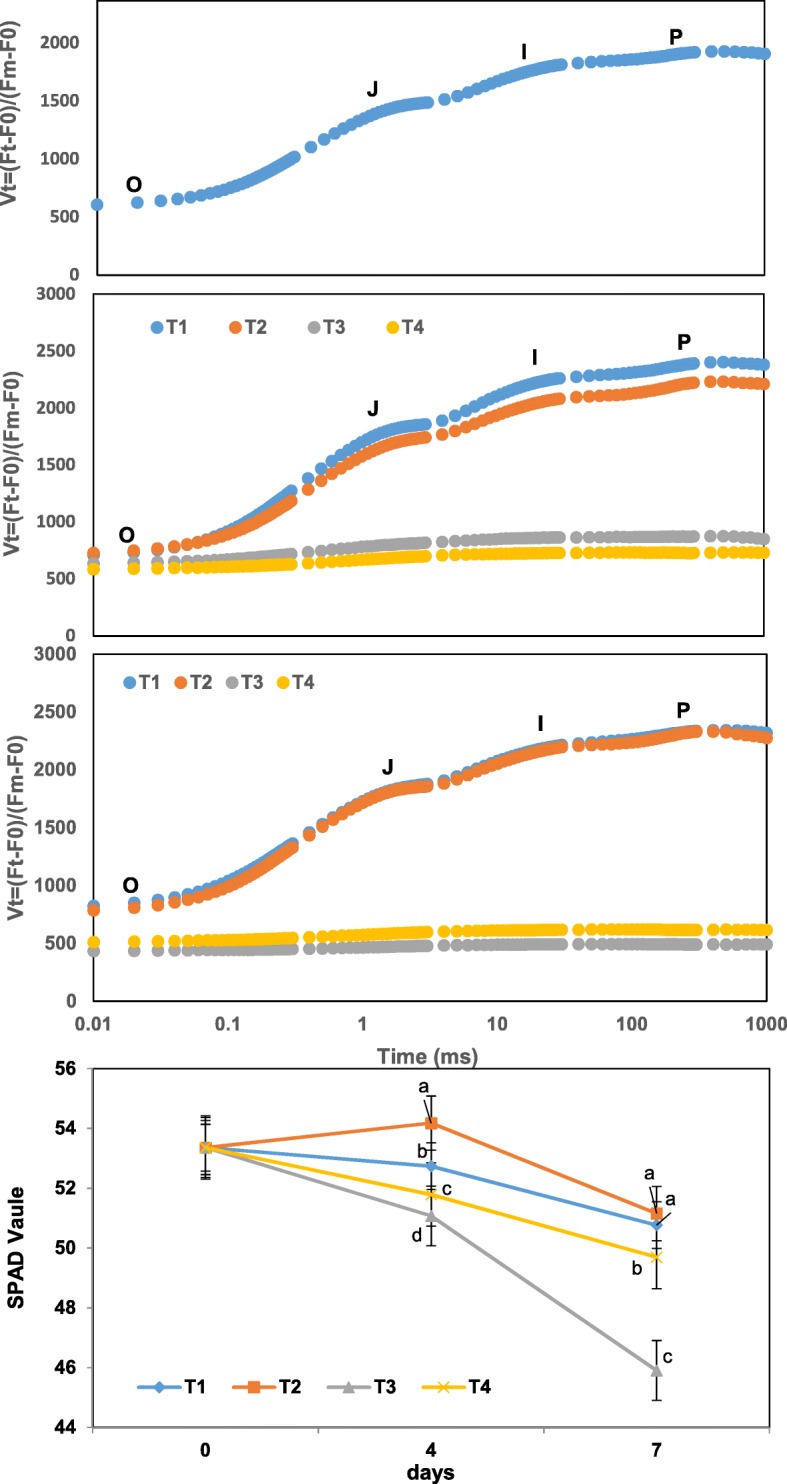
Changes in SPAD value and analysis of tolerance level by chlorophyll fluorescence transients and JIP-test during treatments T1–T4 (25 °C, 25 °C + GABA, 4 °C and 4 °C + GABA for T1, T2, T3 and T4, respectively). Data represent the mean value ± standard deviation. Means with different letters significantly differ from each other (*p* ≤ 0.05)

With no exogenous GABA applied, the SPAD value was significantly lower for low compared to optimum temperature. However, exogenous GABA enhanced SPAD value throughout the experiment (Fig. 2).

Fluorescence transient curves of leaves at optimum temperature showed a typical OJIP shape during days 0–7 without exogenous GABA. Cold treatments showed a much slower growth in the J-step level of the OJIP curves compared with optimum temperature. Conversely, exogenous GABA regardless of temperature led to a significant increase in the J-step level of OJIP curves compared to without GABA. Such sensitivity further confirmed that exogenous GABA improving cold tolerance in tea plants (Fig. 2). Leaf color had no significant change after 7 days with or without GABA, but the leaves were softer under the low temperature for 7 days while others were still hard with exogenous GABA.

### Proteome analysis of leaves in response to treatments

**Fig. 3:**
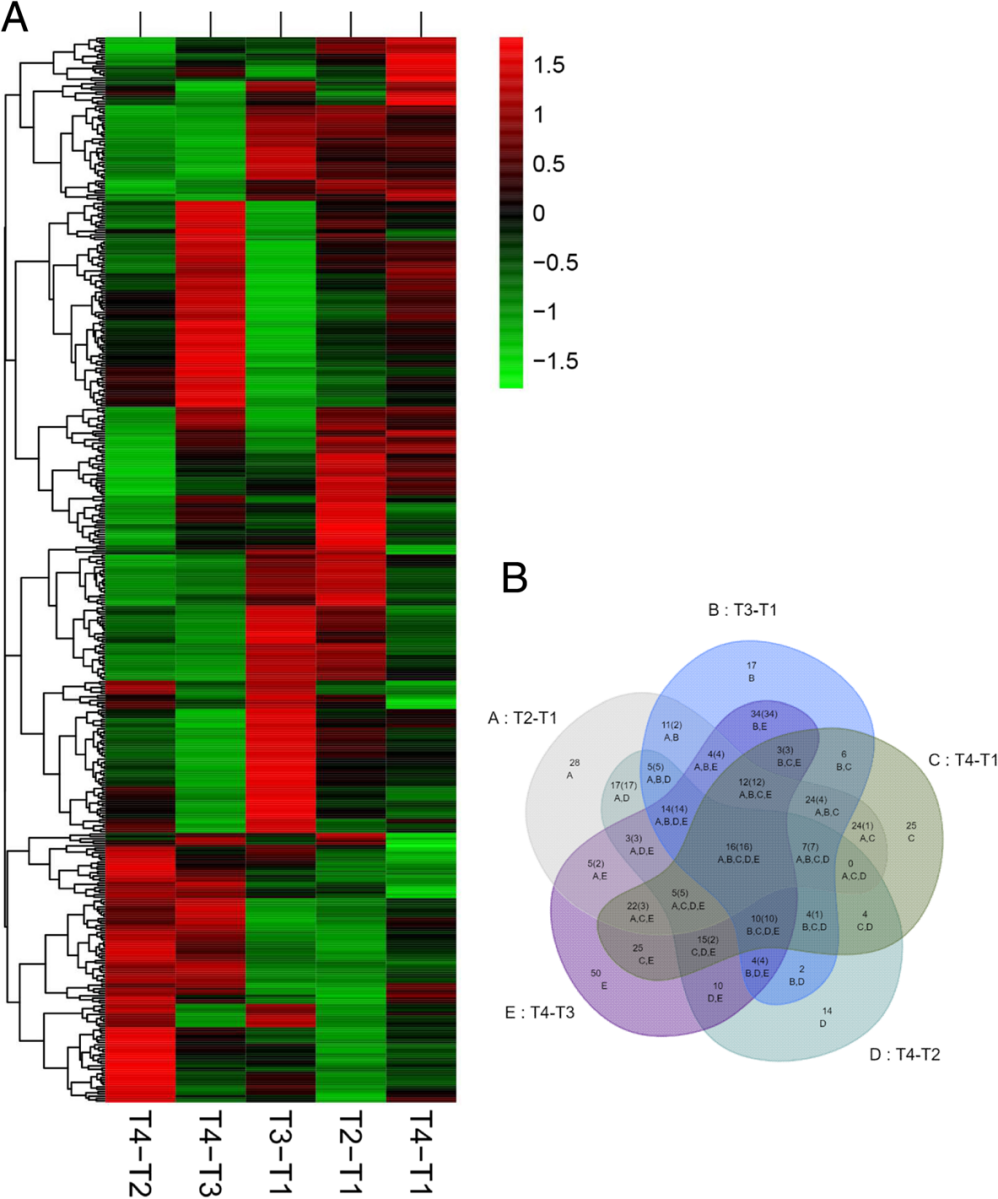
The DAPs in (**a**) clustering analysis and (**b**) the number in leaves of tea plants after exposure to treatments T2/T1, T3/T1, T4/T1, T4/T2 and T4/T3 for 7 days (25 °C, 25 °C + GABA, 4 °C and 4 °C + GABA for T1, T2, T3 and T4, respectively). The color scale bar in the left of hierarchical clustering analysis indicates increased (red) and decreased (green) proteins. Overlapping regions of the circles in the Venn diagram indicate proteins that were regulated in both or all compared treatments, whereas non-overlapping circles indicate proteins regulated in only that treatment

We obtained the proteomes of leaves following exogenous GABA application under low and optimum temperatures, as well as without GABA, to obtain a global view of the molecular responses to the GABA and temperature treatments. To understand proteomic change in the five comparison groups, iTRAQ were applied to determine the protein profiles. According to the criteria (false discovery rate ≤ 0.05 and unique peptide number ≥ 2), 1469 proteins were identified from groups T1–T4. Protein abundances which changed more than 1.5-fold and *p* < 0.05 were regarded as DAPs. Using these criteria, there were 186, 165, 193, 125 and 224 DAPs identified in treatments T2/T1, T3/T1, T4/T1, T4/T2 and T4/T3, respectively. The DAPs from samples of T2 compared to T1 were listed in Additional file 2: Table S2; those from samples of T4 compared to T3 were listed Additional file 3: Table S3; those from samples of T4 compared to T1 were listed in Additional file 4: Table S4. Interestingly, the hierarchical clustering analysis of DAPs in five comparison groups showed that DAPs of T2/T1, T3/T1 (Additional file 5: Table S5), T4/T1, T4/T2 (Additional file 6: Table S6) and T4/T3 had different expression patterns (Fig. 3a). There were 97 common DAPs for T3/T1and T4/T3, 54 for T2/T1 and T4/T3, and 82 for T3/T1and T4/T1 (Fig. 3b). Only 16 DAPs overlapped in all comparison groups. Totals of 28, 17, 25, 14 and 50 DAPs were independently expressed in T2/T1, T3/T1, T4/T1, T4/T2 and T4/T3, respectively (Fig. 3b).

### GO and COG analyses of DAPs in response to exogenous GABA at normal and low temperature

**Fig. 4:**
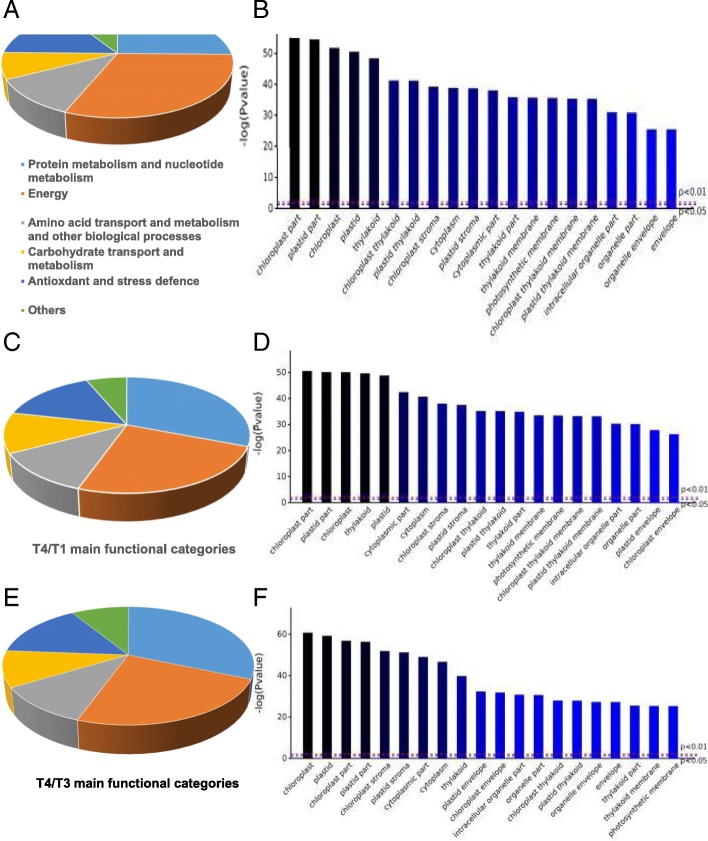
Charts of the DAPs for (**a**) the main functional categories and (**b**) the distribution of proteins according to their cellular localization in tea plant leaves under treatments T2/T1 (25 °C and 25 °C + GABA for T1 and T2, respectively); (**c**) the main functional categories and (**d**) the distribution of proteins according to their cellular localization in tea plant leaves under treatments T4/T1 (25 °C and 4 °C + GABA for T1 and T4, respectively); (**e**) the main functional categories and (**f**) the distribution of proteins according to their cellular localization in tea plant leaves under treatments T4/T3 (4 °C and 4 °C + GABA for T3 and T4, respectively). The tea leaf proteins identified were classified according to their known or predicted cellular localization using Blast2Go (http://www.blast2go.com) program

The proteins corresponding to the identified peptides in tea plants exposed toT2/T1, T4/T1 and T4/T3 were annotated using Blast2GO according to the cell component, biological and molecular function. According to the molecular functions, the DAPs for T2/T1, T4/T1 and T4 /T3 were classified into nine functional categories. For T2/T1, the main functional categories were protein metabolism and nucleotide metabolism (25.3%), energy (30.9%), amino acid transport and metabolism and other biological processes (11.2%), carbohydrate transport and metabolism (7.9%) and antioxidant and stress defense (15.7%) (Fig. 4a). The identified DAPs were mainly predicted to localize in chloroplast part, plastid part, chloroplast, plastid and thylakoid (Fig. 4b). For T4/T1, the main functional categories were protein metabolism and nucleotide metabolism (30.9%), energy (24.5%), amino acid transport and metabolism and other biological processes (11.8%), carbohydrate transport and metabolism (11.3%) and antioxidant and stress defense (15.2%) (Fig. 4c). The DAPs identified were mainly predicted to localize in chloroplast part, plastid part, chloroplast and thylakoid (Fig. 4d). For T4/T3, the main functional categories were protein metabolism and nucleotide metabolism (31.5%), energy (24.0%), amino acid transport and metabolism and other biological processes (10.8%), carbohydrate transport and metabolism (9.9%) and antioxidant and stress defense (15.0%) (Fig. 4e). The identified DAPs were mainly predicted to localize in chloroplast, plastid, chloroplast part and plastid part (Fig. 4f).

### Combined analysis of proteins involved in amino acid transport and contents

**Fig. 5:**
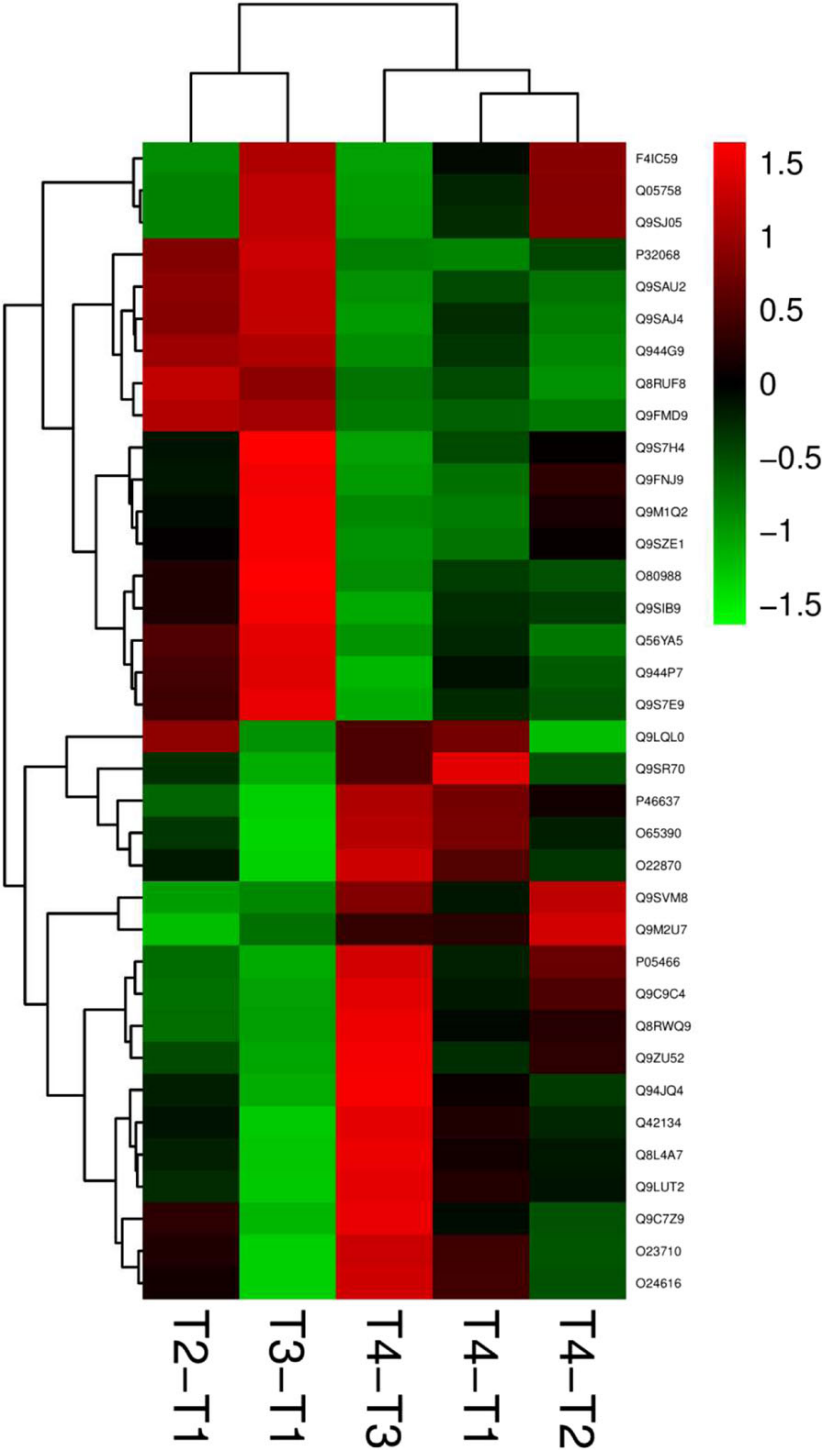
Clustering analysis for DAPs in amino acid transport metabolism in tea leaves after exposure to treatments T2/T1, T3/T1, T4/T1, T4/T2 and T4/T3 for 7 days (25 °C, 25 °C + GABA, 4 °C and 4 °C + GABA for T1, T2, T3 and T4, respectively)

To investigate a possible amino acid transport model for proteins involved in exogenous GABA and temperature, we visualized the expression patterns of amino acid transport-related proteins in response to T2/T1, T3/T1, T4/T3, T4/T1 and T4/T2. There were 36 proteins identified as related to amino acid transport (Fig. 5), some of which had important roles in production of stress response substances and metabolic pathways. Previous study indicated that sk1 (Q9SJ05) was strongly expressed attenuated jasmonate (JA), which forerunner OPDA could stop JA-mediated pistil elimination that protecting a machine-made of sk1 [32]. The significance of ASA1(P32068) in JA-induced auxin synthesis was reported and revealed acharacter for JA attenuated auxin transport in roots and local auxin distribution in fine-tuning root meristems [33]. Liepman [34] concluded that Arabidopsis AGT1 (Q9S7E9) was a peroxisomal photorespiratory enzyme that catalyzed transamination reactions with multiple substrates. Phosphotriglyceride kinase (PGK) transforms 1,3- two phosphate glyceride into 3- phosphate glycolic acid, and participates in the trans reaction of glycolysis and Calvin Benson cycle (CBC) during glycolysis, and the double mutants of PGK3 (Q9SAJ4) and phosphoric acid transporter (PGK3.2-TPT3) exhibited a strong growth phenotype, but feasible [35].

A cut off value of a 1.5-fold-change was employed to strictly assessment a protein as being responsive to T2/T1 compared with T4/T3. The results indicated that amino acid transport in treatments with exogenous GABA under optimum and low temperatures had different expression patterns in tea plants. Comparing treatments T3/T1 with T4/T1, the proteins Glycine-rich RNA-binding protein 2 (Q9SVM8), 3-phosphoshikimate 1-carboxyvinyltransferase (P05466), Enolase 1 (Q9C9C4) and Fructose-bisphosphate aldolase 3 (Q9ZU52) had the same expression patterns; and proteins Aldolase superfamily protein (F4IC59), Leucine aminopeptidase 2 (Q944P7), Thiol protease aleurain-like (Q8RWQ9), Reactive Intermediate Deaminase A (Q94JQ4), Proteasome subunit alpha type-2-B (Q8L4A7) showed no significant differences, but had lower expression in T4 than T3. No proteins showed great differences between treatments T4/T1 and T3/T1; and most of the amino acid transport proteins showed the opposite trend in treatments T4/T1 compared with T3/T1. These results indicated that exogenous GABA under cold stress could redirect amino acid transport toward stress response substances that could relieve stress from low temperature in tea plants. All of the amino acid transport proteins had opposite expression patterns between treatments T3/T1 and T4/T3, which indicated that there were different mechanisms relieving low-temperature stress with or without exogenous GABA at the protein level.

The amino acid contents were measured in the four treatments at 0, 4 and 7 days (Additional file 7: Table S7). Surprisingly, most amino acid contents in tea leaves with exogenous GABA at optimum temperature declined by day 7; However, those at low temperature increased immediately. These results also indicated that amino acid metabolism with exogenous GABA at optimum and low temperatures had completely different expression models. Serine and valine contents in tea leaves in the low compared to optimum temperature treatments declined at day 7 but the other amino acid contents rose. The results implied that application of exogenous GABA raised the amino acid contents and so strengthened plant resistance to low temperature. A previous study summarized GABA accumulates rapidly in stress tissue and might supply a key link for event chain that perceives physiological response from environmental pressure. [15] These results indicate that exogenous GABA may lead to transport of amino acids to take part in metabolic pathways to adapt to low temperature.

### Interaction network analysis of response to exogenous GABA at low temperature

**Fig. 6:**
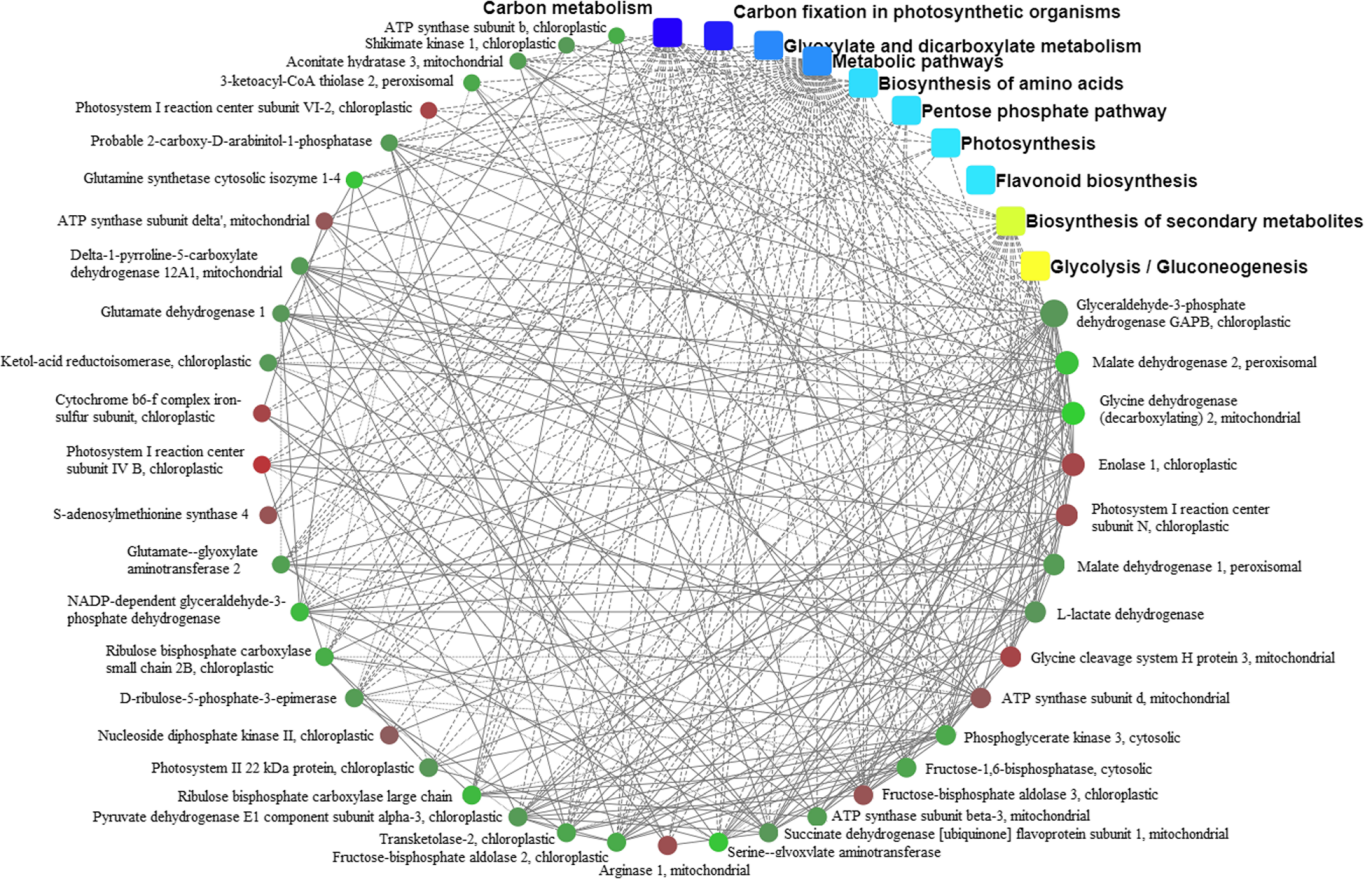
Interaction network of DAPs in tea leaves exposed to treatments T4/T3 (4 °C and 4 °C + GABA for T3 and T4, respectively). Pathways and interactions connected to all of the DAPs analyzed using Pathway Studio software. Notes: Dots represents proteins, red represents increased expression and green represents decreased expression. The rounded rectangles represent biological processes, cell localization, molecular function or signaling pathway; blue represents significantly higher and yellow represents less significance. Lines represent a relationship between each other; solid lines represent a relationship with the relevant verification, and dotted lines represent unverified

Proteins do not perform their functions in cells as single entities, but act in a network. [36] There were 47 proteins identified in this interaction network (Fig. 6, Additional file 8: Table S8, S9 & S10). These proteins were classified functionally into carbon fixation in photosynthetic organisms (13 proteins), glyoxylate and dicarboxylate metabolism (11), biosynthesis of amino acids (17), pentose phosphate pathway (8), flavonoid biosynthesis (4), glutathione metabolism (6), TCA cycle (5), ascorbate and aldarate metabolism (4) and purine metabolism (2). These may be critical components in the response to low temperature between with and without exogenous GABA in the tea plants. For example, purple-leaf tea plants are anthocyanin-rich cultivars that are valuable materials for manufacturing teas with unique colors or flavors; and metabolites in the flavonoid biosynthetic pathway remain at high levels in purple leaves [37]. Flavonol synthase can be silenced or overexpressed to shunt the flavonoid biosynthetic pathway toward anthocyanin production without adverse effects on plant growth and development [38]. ACO (1-aminocyclopropane-1-carboxylate oxidase) isoforms might be necessary for plants to synthesis ethylene under various abiotic environment [39].

## Discussion

### Physiological and proteomic mechanisms with different temperatures and exogenous GABA through GABA shunt

Plants have evolved complex mechanisms to deal with low temperature. When plants sense low temperature, a series of protective mechanisms are triggered – including decreasing malondialdehyde (MDA) content, which is considered to be an indicator of plant oxidative stress; synthesizing cryoprotectant molecules and low-molecular-weight nitrogenous compounds; and improving the scavenging activity of reactive oxygen species (ROS) [40–42]. In addition, chlorophyll fluorescence imaging can be employed to evaluate cold tolerance in numerous plants. [43] These alterations help plants keep a metabolic balance of substance and energy in cold environments. Under low temperature, tea plants adopted a ‘survival mode’ as shown by higher free proline content, decreased MDA content, regulating the antioxidant activity and reduced photosynthesis, resulting in growth arrest (Additional file 9: Figure S1 and Additional file 10: Figure S2). GABA can be quickly accumulated and is involved in adapting to low-temperature stress [44, 45]. However, in our study, the endogenous GABA content decreased when plants were exposed to low temperature. We speculate that endogenous GABA degraded to promote synthesis of other stress response substances and feedback to the TCA cycle at low temperature, which showed that other amino acid contents and the DAPs in the TCA cycle were up-regulated at low compared to optimum temperature.

The GABA metabolism in tea plants could regulate the GABA precursor glutamate and PAs contents to relieve anoxia stress in our previous study [14]. No significant changes in GABA-T, GAD and DAO activities, Put and Spm levels for the two different temperatures compared to controls, while application of exogenous GABA. The PAO activity clearly decreased compared to control for optimum temperature at day 4, and the SPD concentration was higher at low temperature than the control (Additional file 11: Figure S3 and Additional file 12: Figure S4). The results imply that application of exogenous GABA can feed back to the TCA cycle to promote the carbon and nitrogen cycle. The amino acid contents except for alanine and lysine all declined (Additional file 7: Table S7). A reversible transformation of an amino acid from glutamic acid to pyruvic acid was catalyzed by alanine aminotransferase (AlaAT) form 2-oxoglutarate and alanine. The AlaAT-related protein (Q9S7E9) showed no significant change in treatment T2 compared to T1, which matched the report showing that AlaAT1 broke down excess alanine [46]. Lysine is synthesized by a special branch of the family pathway of aspartic acid. Carbon atoms from lysine to acetyl coenzyme A, which then enters the TCA cycle, generatingα-ketoglutaric acid [47]. These results imply that, at optimum temperature, application of exogenous GABA influenced the GABA level resulting in response of metabolism pathways to the alert in carbon and nitrogen transport.

The iTRAQ-based protein analysis was a great information-rich approach for hunting the stress-induced dynamic proteins; for pinning down the key metabolic pathways, it may play important roles in the response of improving resistance to low temperature in tea plants with application of exogenous GABA. The results indicated that application of exogenous GABA at low temperature compared to control profoundly changed metabolic pathways, including amino acids biosynthesis, flavonoid biosynthesis, glyoxylate and dicarboxylate metabolism, carbon fixation in photosynthetic organisms and the pentose phosphate pathway. These pathways included most of the DAPs in treatments T4/T3; however, pathways such as TCA cycle, glutathione metabolism, ascorbate and aldarate metabolism, and purine metabolism were also important. To our knowledge, this is the first report on dynamic proteomic responses to exogenous GABA application at low temperature in tea plants.

### Flavonoid metabolism regulated with exogenous GABA at low temperature

**Fig. 7:**
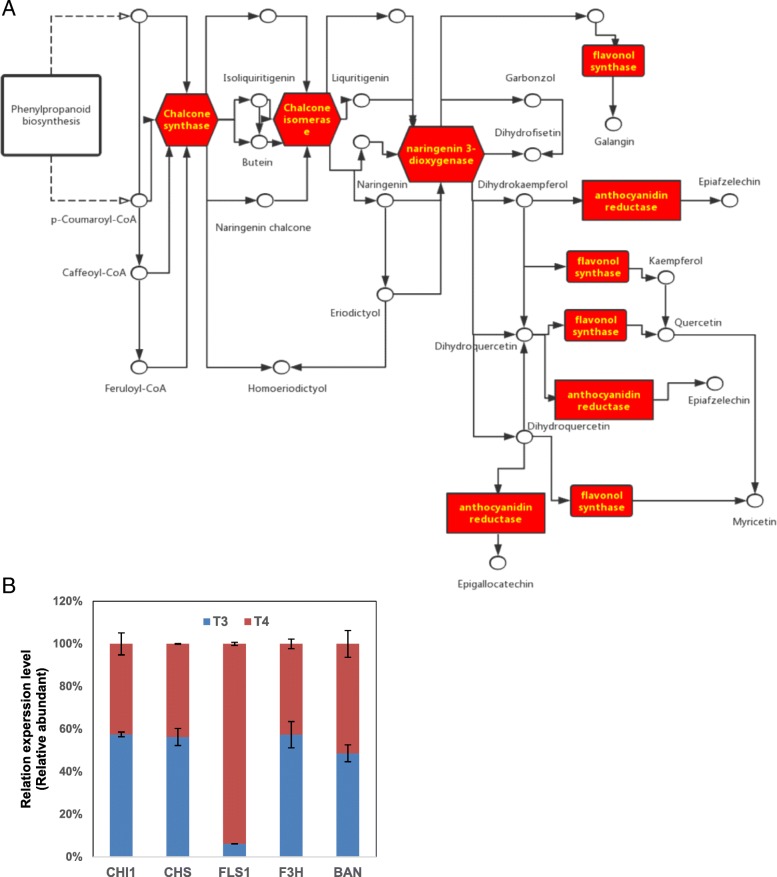
Effect of application of exogenous GABA at low temperature on (**a**) DAPs and (**b**) RT-qPCR analysis of genes related to flavonoid biosynthesis. Transcript abundance was calculated according to the difference in cycle threshold values between the target gene and *β-actin* transcripts normalized by the 2^−ΔΔCT^ method. The mRNA levels of the genes in tea leaves at 0 h were set as 1.0. Data represent the mean value ± standard deviation. Means with different letters significantly differ from each other (*p* ≤ 0.05). *BAN:* NAD(P)-binding Rossmann-fold superfamily protein; *CHI1:* chalcone flavanone isomerase 1; *CHS:* chalcone synthase; *F3H:* flavanone 3-hydroxylase; *FLS1:* flavonol synthase 1

Flavonoids are important secondary metabolites in plants and play important roles in many functions, which included pigment and antioxidant activities. Five DAPs related to flavonoid biosynthesis were observed in treatment T4/T3 (Fig. 7a). Anthocyanins are a major class of flavonoids, whose functions are very diverse which include antioxidant activity, UV rejection, defense of plant pathogens, leguminous nodulation, man fertility, optical signal and auxin transport govern [48]. Under salt treatments, the proline content increased while the wheat genotypes have higher anthocyanin content [49]. Low temperature induces anthocyanin synthesis in various species [50], it was also reported that overexpression of flavonol glycosides and anthocyanins in plants under abiotic stress can effectively remove ROS and improve drought tolerance of plants. [51, 52] Of the three anthocyanin-related genes selected for RT-qPCR, *CsBAN* was up-regulated and *CsCHS* and *CsF3H* were down-regulated (Fig. 7b), which indicating that the expression of *CsCHS*, *CsF3H* and *CsBAN* involved in anthocyanin biosynthesis, could be induced by cold treatment [53]. Our results also indicate that the effects of exogenous GABA for the three genes involved in anthocyanin synthesis in tea plants at low temperature differed, as did the genes in the process of mRNA expression and protein expression in the flavonoid metabolism, and should be further studied.

### Amino acid metabolism and ascorbate (AsA)/glutathione cycle and exogenous GABA at low temperature

The levels of GABA, proline, tryptophan, histidine, asparagine and alanine showed the greatest changes in abundance in response to GABA application at low temperature compared to control (Additional file 7: Table S7). Combining with the metabolome and proteome, we found no significant relation between them. There are few published studies of amino acid export in the xylem under saline conditions [54]. After being treated with exogenous GABA at low temperature for 7 days, except for glycine, contents of all amino acids in leaves rose greatly.

**Fig. 8:**
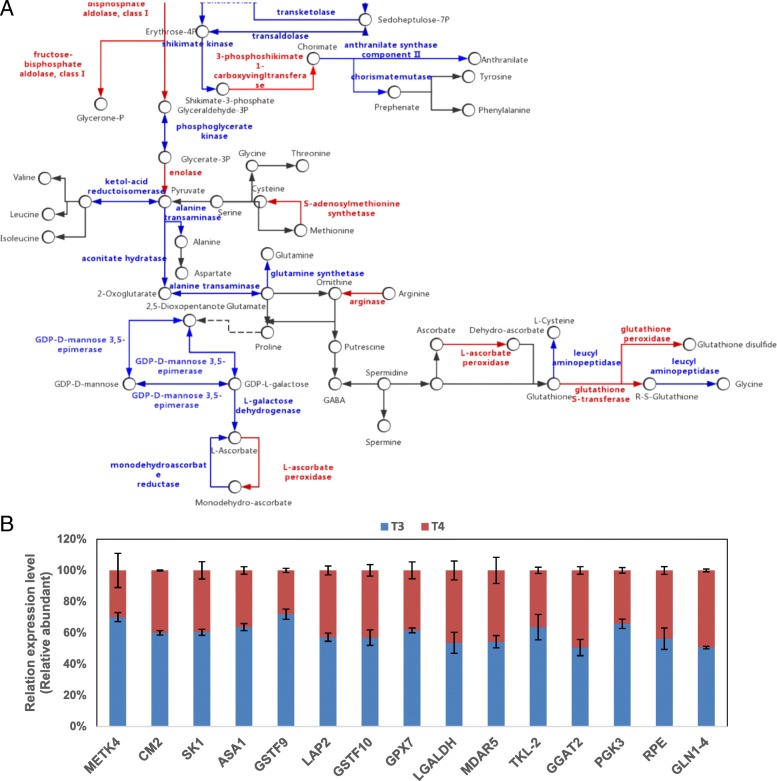
Effect of application of exogenous GABA at low temperature on (**a**) DAPs and (**b**) RT-qPCR analysis of genes related to amino acid metabolism and AsA/glutathione cycle. Transcript abundance was calculated according to the difference in cycle threshold values between the target gene and *β-actin* transcripts normalized by the 2^−ΔΔCT^ method. The mRNA levels of the genes in tea leaves at 0 h were set as 1.0. Data represent the mean value ± standard deviation. Means with different letters significantly differ from each other (*p* ≤ 0.05). Abbreviations: *ASA1:* anthranilate synthase alpha subunit 1; *CM2:* chorismate mutase 2; *GGAT2:* glutamate--glyoxylate aminotransferase 2-like; *GLN1–4:* glutamine synthetase 1–4; *GPX7:* glutathione peroxidase 7; *GSTF10:* glutathione S-transferase PHI 10; *GSTF9:* glutathione S-transferase PHI 9; *LAP2:* Cytosol aminopeptidase family protein; *LGALDH:* L-galactose dehydrogenase; *MDAR5:* monodehydroascorbate reductase 5, chloroplastic-like; *METK4:* S-adenosylmethionine synthetase 4; *PGK3:* phosphoglycerate kinase precursor; *RPE:* ribulose-5-phosphate-3-epimerase; *SK1:* shikimate kinase 1; *TKL-2:* transketolase 2

Glutamine synthetase (GS) is an enzyme in the process of plant primary nitrogen assimilation, since glutamine and other related nitrogenous compounds are converted by it catalyzing ammonium, which is also a substrate for protein synthesis. The reabsorption of ammonia in photorespiration is the main role of chloroplast GS, and chloroplastic GS aided nitrogen assimilation in chloroplasts of YL [55]. GS was down-regulated here, and most amino acid contents increased, indicating that GS played an important role in the process that GABA works as a signal molecule when plants exposed to cold stress. Cysteine synthase catalyzes the synthesis of cysteine from O-acetylserine and disulfides, which serves as the only amino acid containing disulfide bonds (S–S) that protecting cellular environments from oxidative stress [55]. Cysteine synthase and spermidine synthase might be associated to the rise molecular chaperone activity and molecular chaperone and the decline in oxidative stress [56]. In the present study, cysteine and spermidine contents increased significantly but no differences between treatment T4 and T3, suggesting regulatory mechanisms occurring in response to cysteine and spermidine and involving both transcriptional and post-translational levels (Fig. 8a).

Cell process and in vitro stress produce AsA, a step in the biosynthetic pathway of which is the catalysis of GDP-mannose-3′, 5′-diisomerase (GME) between GDP-galactose and GDP-mannose [57]. Spinach L-galactose dehydrogenase (L-GalDH) show invertible inhibition by AsA, the final product of biosynthetic pathway [58]. AsA peroxidase (APX) plays a main role in cellular H_2_O_2_ metabolism. GME, L-GalDH and leucyl amino peptidase had lower expression in tea plants, but APX, glutathione S-transferase and glutathione exhibited an opposite trend (Fig. 8a), which implied that exogenous GABA application might regulate AsA and glutathione metabolism by different mechanisms. Sensitive genes in amino acid metabolism and the AsA/glutathione cycle had their relative expressions determined using RT-qPCR (Fig. 8b) and these were consistent with the related DAPs.

### TCA cycle, glyoxylate cycle and carbon fixation in photosynthetic organs and GABA shunt and exogenous GABA at low temperature

**Fig. 9:**
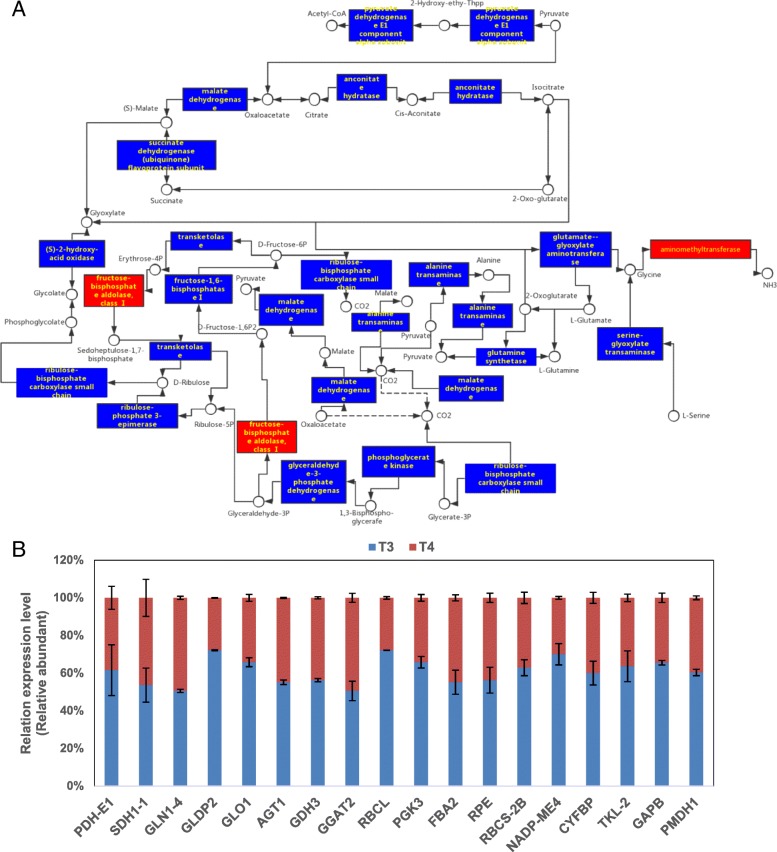
Effect of application of exogenous GABA at low temperature on (**a**) DAPs and (**b**) RT-qPCR analysis of genes related to TCA cycle, glyoxylate cycle and carbon fixation in photosynthetic organisms. Transcript abundance was calculated according to the difference in cycle threshold values between the target gene and *β-actin* transcripts normalized by the 2^−ΔΔCT^ method. The mRNA levels of the genes in tea leaves at 0 h were set as 1.0. Data represent the mean value ± standard deviation. Means with different letters significantly differ from each other (*p* ≤ 0.05). Abbreviations: *AGT1:* alanine-glyoxylate transaminase; *FBA2:* fructose-bisphosphate aldolase 2; GAPB: glyceraldehyde-3-phosphate dehydrogenase B subunit; *GDH3:* glutamate dehydrogenase 3; *GGAT2:* glutamate--glyoxylate aminotransferase 2-like; *GLDP2:* glycine decarboxylase P-protein 2; *GLO1:* glyoxalase; *NADP-ME4:* NADP-malic enzyme 4; *PDH-E1:* pyruvate dehydrogenase E1; *PMDH1:* peroxisomal NAD-malate dehydrogenase 1; *RBCL:* ribulose-1,5-bisphosphate carboxylase/oxygenase large subunit; *RBCS-2B:* Ribulose bisphosphate carboxylase small chain 2B; *SDH1–1:* succinate dehydrogenase 1–1

The TCA cycle includes important approaches to plant synthetic amino acid, energy supply and different biological movement course [59]. Malate dehydrogenase (MDH) catalyzes the effect of NAD+ on NADH oxidation of malic acid was reproduced in mitochondrial matrix during TCA cycle [60]. Environmental stresses including drought, heat, salinity and aluminum can decrease the MDH level in various plant species [61–63]. Exogenous GABA application at low temperature resulted in lower MDH level compared with the control, suggesting that exogenous GABA improved the low-temperature-induced reduction in MDH to catalyze the inhibited malate to oxaloacetate during malate metabolism. All of the DAPs in the TCA cycle showed greater down-regulation in treatment T4 compared to T3 (Fig. 9a).

In addition, MDH participates in amino acid synthesis because of the reactions of malic acid, acetoacetic acid and aspartic acid [64]. With the significant decrease in MDH and succinate dehydrogenase and increases in aspartic acid, the contents of threonine, isoleucine, lysine, alanine, valine and serine were elevated with exogenous GABA application at low temperature. It was reported that the levels of alanine, valine and serine were affected by the stimulation of elevated endogenous GABA under abiotic stresses: Bermuda grass under heat stress [65] and tobacco under salt [66]. The GABA shunt was considered to be a part of the TCA cycle during respiration [67–69], which is also important in primary carbon and nitrogen metabolism [70]. Increased GABA promoted the alanine substance accumulation, which entered the proline metabolism and TCA cycle. All amino acid contents except for glycine were increased by elevated GABA at low temperature, which suggested that GABA increased the carbon and nitrogen metabolism. However, the proteins related to the GABA shunt showed no significant changes, but the GABA precursors and the enzyme activity of GABA metabolism clearly changed.

Glyoxylate cycle was firstly discovered in microorganisms, which provided substrates for biosynthetic processes and respiration [71]. In addition to lipids, glyoxylate cycle is another carbon source necessary for growth after germination [72]. Most of the proteins involved in the glyoxylate cycle were down-regulated in response to low temperature with exogenous GABA compared to the control. Furthermore, the exogenous GABA could relieve the cold damage to photosystem II, which was crucial in carbon metabolic [73]. There are many signaling molecules, such as nitrate, ammonium, sugar, amino acids and organic acids, also contain interactions between carbon and nitrogen metabolism [74]. Nitrogen uptake and metabolism is contributed by carbon metabolism since it requires carbon skeletons, reducing power, ATP, reductants and co-transporters. [73, 75–77]. Their control may be closely linked and coordinated at the level of gene expression [78]. However, only aminomethyl transferase and fructose-bisphosphate aldolases (FBAs) were up-regulated in the TCA cycle, glyoxylate and dicarboxylate metabolism, and carbon fixation in photosynthetic organisms in treatment T4 compared to T3. Fructose 1,6-bisphosphate aldolases, which are important in the Calvin-Benson cycle (CBC), were dramatically altered when tomato seedlings suffer from heat/cold stresses [79]. This might imply that exogenous GABA application could improve photosynthesis to regulate low-temperature response through *FBA* expression and enzyme activity.

The above evidence suggests that, as a signal molecule, GABA regulates the glyoxylate cycle, the TCA cycle and carbon fixation in photosynthetic organ metabolism in a complex manner, especially in the carbon and nitrogen cycle. It will be interesting to establish how tea plants respond to low temperature via different mechanisms and how the signal molecule GABA influences the operating mechanisms. Several genes in the TCA cycle, glyoxylate cycle and carbon fixation in photosynthetic organisms had their relative expression determined by RT-qPCR (Fig. 9b) and the expression mode of genes was similar to the response of related DAPs.

### Oxidative pentose phosphate pathway and purine metabolism and exogenous GABA at low temperature

The energy and metabolic intermediates in the biosynthesis are mainly derived from the pentose oxidation pathway. However, further details are needed on how the pathway and its effects on other processes in plants. Nitrate is the major source of nitrogen that plants need, and the location of nitrate assimilation significantly affect the plant energy budget [73]. This implies that exogenous GABA application could affect the energy budget in tea plants. Only FBA in the oxidative pentose phosphate pathway was up-regulated in treatment T4 compared to T3 here. Lu et al. [80] reported that all *FBA* genes in *Arabidopsis thaliana*, except *AtFBA6*, were up-regulated in response to cold stress. The carbon skeleton of the amino acid synthesis pathway arises from different sectors of the respiratory pathway. Some root in the oxidative pentose phosphate pathway (or, in the light, the CBC) and glycolysis (Ery4P and PEP for the synthesis of aromatic amino acids), some stem from the final product of glycolysis (pyruvate for alanine) and other organic acids from the TCA cycle [73]. For treatment T4 compared to T3, FBA was not only in glycolysis cycle but also in CBC, *FBA2* and *FBA3* genes were up-regulated, but the DAPs in the TCA cycle were down-regulated. Testing how the application of exogenous GABA in tea plants affects the respiration pathway of amino acid biosynthesis through reverse genetic pathway under low temperature, is further needed.

**Fig. 10:**
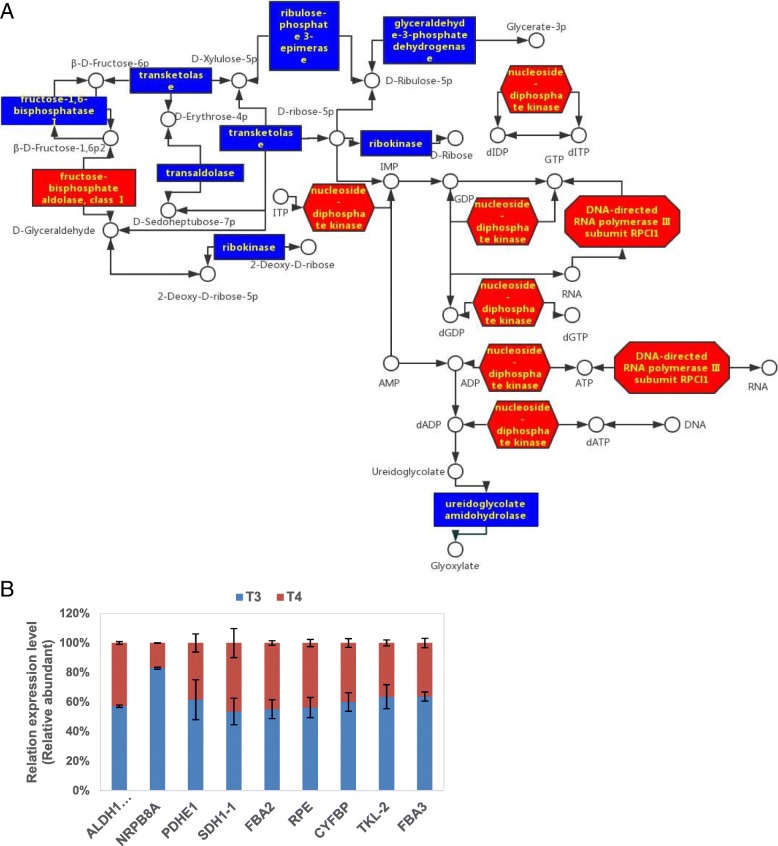
Effect of application of exogenous GABA at low temperature on (**a**) DAPs and (**b**) RT-qPCR analysis of genes related to oxidative pentose phosphate pathway and purine metabolism. Transcript abundance was calculated according to the difference in cycle threshold values between the target gene and *β-actin* transcripts normalized by the 2^−ΔΔCT^ method. The mRNA levels of the genes in tea leaves at 0 h were set as 1.0. Data represent the mean value ± standard deviation. Means with different letters significantly differ from each other (*p* ≤ 0.05). Abbreviations: *ALDH1:* Aldehyde dehydrogenase; *CYFBP*: Fructose-1,6-bisphosphatase; *FBA3:* fructose-1, 6-bisphosphate aldolase

Purine nucleotides are produced by two distinct routes in plants: de novo and salvage pathways. The de novo synthesis employs 5-phosphoribosyl-1-pyrophosphate, aspartate, glycine, glutamine, HCO^3−^ and 10-formyltetrahydrofolateas for building blocks, which could be found in all plants, such as tea-leaves; however, the salvage pathways are more diverse and less well understood [81, 82]. Tea plants engender special nitrogen compounds including theanine and caffeine and their effects on human health have been studied in detail [83]. In order to against herbivores and pathogens, young shoots will accumulate caffeine and leaves may be treated as a chemical defense of young soft tissue [84]. All the DAPs in purine metabolism, except ureidoglycolate amidohydrolase, were up-regulated (Fig. 10a).

**Fig. 11:**
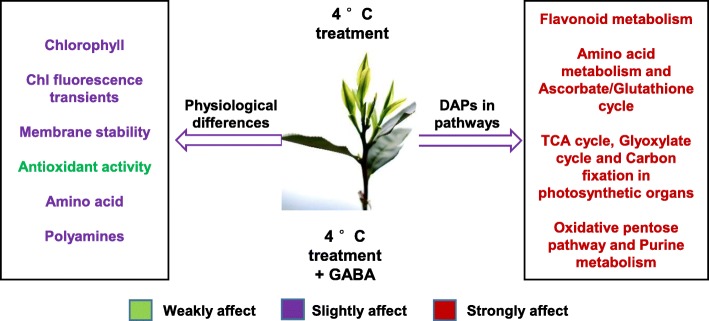
Effect of exogenous GABA at low temperature on physiological index and DAPs in metabolism pathways. Models of physiological index and possible metabolism pathways by exogenous GABA compared to that without GABA under low temperature in the tea plants. Antioxidant activities had no significant difference (green), Polyamine contents increased slightly (blue), while other index and the metabolism pathways above were affected by exogenous GABA (red)

Thus, the evidence suggests that exogenous GABA application either directly or indirectly stimulated flux into amino acid and caffeine biosynthesis and regulated the plant energy budget. This affected the resistance to cold in tea plants and the quality of tea flavor through the oxidative pentose phosphate pathway and purine metabolism. Key genes in the oxidative pentose phosphate pathway and purine metabolism were selected for RT-qPCR, which showed that expression mode of genes were similar to that of related DAPs (Fig. 10b). Above all, the effects of exogenous GABA at low temperature on physiological index and DAPs in metabolism pathways, were summarized on Fig. 11.

## Conclusions

GABA effectively improved tea plant tolerance at low temperature and regulated various physio-biochemical processes at optimum condition, as reflected by higher SPAD value, chlorophyll fluorescence transients and membrane stability, as well as antioxidant activity regulation. The iTRAQ-based protein profiling offered a global road to show metabolic processes related to plant tolerance which was regulated by endogenous GABA at low temperature. Not only the transformation of endogenous GABA is further demonstrated in functional protein networks, but also is revealed in metabolism pathways, such as flavonoid metabolism, amino acid metabolism, AsA/glutathione cycle, TCA cycle, glyoxylate cycle, carbon fixation in photosynthetic organisms, oxidative pentose phosphate pathway and purine metabolism cause an interaction among plant cell components like chloroplasts, plastids and thylakoids. How GABA works as a signaling molecule involving in tea plants resistance regulation under cold stress, may be an interesting topic in the future work.

## Additional files

Additional file 1: **Table S1:** Nucleotide sequences of primers specific to flavonoid biosynthesis, amino acid metabolism and AsA/glutathione cycle, TCA cycle, glyoxylate cycle and carbon fixation in photosynthetic organisms and oxidative pentose phosphate pathway and purine metabolism related genes used for qRT-PCR. (XLSX 10 kb)

Additional file 2: **Table S2:** Functional classifications of identified proteins significantly expressed in leaves of tea plants exposed to the treatments T2/T1 for 7 days (25 °C and 25 °C + GABA for T1 and T2 respectively). Each value represents the average of three biological replicates. The average is significant at a *p* < 0.01 level. A t-test value < 0.05 is considered to be significant between treatment T2 and T1. (XLSX 41 kb)

Additional file 3: **Table S3:** Functional classifications of identified proteins significantly expressed in leaves of tea plants exposed to the treatments T4/T3 for 7 days (4 °C and 4 °C + GABA for T3 and T4, respectively). Each value represents the average of three biological replicates. The average is significant at a *p* < 0.01 level. A t-test value < 0.05 is considered to be significant between treatment T4 and T3. (XLSX 47 kb)

Additional file 4: **Table S4:** Functional classifications of identified proteins significantly expressed in leaves of tea plants exposed to the treatments T4/T1 for 7 days (25 °C and 4 °C + GABA for T1 and T4, respectively). Each value represents the average of three biological replicates. The average is significant at a *p* < 0.01 level. At-test value < 0.05 is considered to be significant between treatment T4 and T1. (XLSX 40 kb)

Additional file 5: **Table S5:** Functional classifications of identified proteins significantly expressed in leaves of tea plants exposed to the treatments T3/T1 for 7 days (25 °C and 4 °C for T1 and T3, respectively). Each value represents the average of three biological replicates. The average is significant at a *p* < 0.01 level. A t-test value < 0.05 is considered to be significant between treatment T3 and T1. (XLSX 32 kb)

Additional file 6: **Table S6:** Functional classifications of identified proteins significantly expressed in leaves of tea plants exposed to the treatments T4/T2 for 7 days (25 °C + GABA and 4 °C + GABA for T2 and T4, respectively). Each value represents the average of three biological replicates. The average is significant at a *p* < 0.01 level. At-test value < 0.05 is considered to be significant between treatment T4 and T2. (XLSX 27 kb)

Additional file 7: **Table S7:** The contents of amino acids composition in tea plant leaves in the treatments T1, T2, T3, T4 at day 0, 4, 7 (25 °C, 25 °C + GABA, 4 °C and 4 °C + GABA for T1, T2, T3 and T4, respectively). All experiments were performed in triplicate. Data represent the mean value ± standard deviation. Means with different letters are significantly different fromeach other (*p* ≤ 0.05). Asp, Aspartic acid; Ser, Serine; Glu, Glutamate; Gly, Glycine; Ala, Alanine; Cys, Cysteine; Val, Valine; Met, Methionine; Ile, Isoleucine; Leu, Leucine; Tyr, Tyrosine; Phe, Phenylalanine; GABA, Gama-aminobutyric acid; Lys, Lysine; His, Histidine; Trp, Tryptophane; Arg, arginine. (DOCX 15 kb)

Additional file 8: **Table S8**, **S9 & S10:** Sequences of all unique peptides matched to each identified proteins. (ZIP 1383 kb)

Additional file 9: **Figure S1:** Changes in free proline content and MDA activity during the four treatments (25 °C, 25 °C + GABA, 4 °C and 4 °C + GABA for T1, T2, T3 and T4, respectively). All experiments were performed in triplicate. Data represent the mean value ± standard deviation. Means with different letters are significantly different from each other (*p* ≤ 0.05). MDA, malonaldehyde. (PDF 153 kb)

Additional file 10: **Figure S2:** Changes in antioxidant enzyme activity during the four treatments (25 °C, 25 °C + GABA, 4 °C and 4 °C + GABA for T1, T2, T3 and T4, respectively). Data represent the mean value ± standard deviation. Means with different letters are significantly different from each other (*p* ≤ 0.05). CAT, catalase; SOD, superoxide dismutase; POD, peroxidase. (PDF 167 kb)

Additional file 11: **Figure S3:** Changes in DAO, PAO, GAD and GABA-T activity. Data represent the mean value ± standard deviation. Means with different letters are significantly different from each other (*p* ≤ 0.05). DAO, diamine oxidase; PAO, polyamine oxidase; GAD, glutamate decarboxylase; GABA-T, GABA transaminase. (PDF 170 kb)

Additional file 12: **Figure S4:** Changes in polyamines contents during the four treatments (25 °C, 25 °C + GABA, 4 °C and 4 °C + GABA for T1, T2, T3 and T4, respectively). Data represent the mean value ± standard deviation. Means with different letters are significantly different from each other (*p* ≤ 0.05). Put, putrescine; Spd, spermidine; Spm, spermine. (PDF 158 kb)

## Abbreviations

ABA: Abscisic acid
ACO: 1-Amino cyclopropane carboxylic acid oxidase
*AGT1*: Alanine-glyoxylate transaminase
AlaAT: Alanine aminotransferase
*ALDH1*: Aldehyde dehydrogenase
APX: AsA peroxidase
ASA1: anthranilate synthase alpha subunit 1
BAN: NAD(P)-binding Rossmann-fold superfamily protein
CAT: Catalase
CBC: Calvin-Benson cycle
*CHI1*: Shalcone flavanone isomerase 1
*CHS*: Chalcone synthase
*CM2*: Chorismate mutase 2
*CYFBP*: Fructose-1,6-bisphosphatase
DAO: Diamine oxidase
DAPs: Differentially abundant proteins
*F3H*: Flavanone 3-hydroxylase
FBA: Fructose-1,6-bisphosphate aldolase
*FBA2*: Fructose-bisphosphate aldolase 2
*FLS1*: Flavonol synthase 1
GABA: Gamma-aminobutyric acid
GABAT: Gamma aminobutyrate transaminase
GAD: Glutamate decarboxylase
GAPB: Glyceraldehyde-3-phosphate dehydrogenase B subunit
*GDH3*: Glutamate dehydrogenase 3
*GGAT2*: Glutamate--glyoxylate aminotransferase 2-like
*GLDP2*: Glycine decarboxylase P-protein 2
*GLN1–4*: Glutamine synthetase 1–4
*GLO1*: Glyoxalase
GME: GDP-mannose-3′, 5′-diisomerase
*GPX7*: Glutathione peroxidase 7
GS: Glutamine synthetase
*GSTF10*: Glutathione S-transferase PHI 10
*GSTF9*: Glutathione S-transferase PHI 9
H_2_O_2_: Hydrogen peroxide
iTRAQ: Isobaric tags for relative and absolute quantification
*LAP2*: Cytosol aminopeptidase family protein
*LGALDH*: L-galactose dehydrogenase
MDA: Malondialdehyde
*MDAR5*: Monodehydroascorbate reductase 5, chlorplastic-like
MDH: Malate dehydrogenase
*METK4*: S-adenosylmethionine synthetase 4
*NADP-ME4*: NADP-malic enzyme 4
PAO: Polyamine oxidase
PAs: Polyamines
*PDH-E1*: Pyruvate dehydrogenase E1
PEP: Phosphoenolpyruvate
PGK: Phosphotriglyceride kinase
*PGK3*: Phosphoglycerate kinase precursor
PGK3.2-TPT3: phosphoric acid transporter
*PMDH1*: Peroxisomal NAD-malate dehydrogenase 1
POD: Peroxidase
Put: Putrescine
*RBCL*: Ribulose-1,5-bisphosphate carboxylase/oxygenase large subunit
*RBCS-2B*: Ribulose bisphosphate carboxylase small chain 2B
ROS: Reactive oxygen species
*RPE*: Ribulose-5-phosphate-3-epimerase
SDH: Succinate dehydrogenase
*SDH1–1*: succinate dehydrogenase 1–1
*SK1*: Shikimate kinase 1
SOD: Superoxide dismutase
Spd: Spermidine
Spm: Spermine
TCA cycle: Tricarboxylic acid cycle
*TKL-2*: Transketolase 2
