# Supplementary material for: Physiological and iTRAQ-based proteomic analyses reveal the function of exogenous γ-aminobutyric acid (GABA) in improving tea plant (Camellia sinensis L.) tolerance at cold temperature
Source: BMC Plant Biol. 2019 Jan 30;19:43. doi: 10.1186/s12870-019-1646-9 (PMC6354415; doi:10.1186/s12870-019-1646-9)
Supplement: Supplementary file 7 — Table S7: The contents of amino acids composition in tea plant leaves in the treatments T1, T2, T3, T4 at day 0, 4, 7 (25 °C, 25 °C + GABA, 4 °C and 4 °C + GABA for T1, T2, T3 and T4, respectively). All experiments were performed in triplicate. Data represent the mean value ± standard deviation. Means with different letters are significantly different fromeach other (p ≤ 0.05). Asp, Aspartic acid; Ser, Serine; Glu, Glutamate; Gly, Glycine; Ala, Alanine; Cys, Cysteine; Val, Valine; Met, Methionine; Ile, Isoleucine; Leu, Leucine; Tyr, Tyrosine; Phe, Phenylalanine; GABA, Gama-aminobutyric acid; Lys, Lysine; His, Histidine; Trp, Tryptophane; Arg, arginine. (DOCX 15 kb) [file 12870_2019_1646_MOESM7_ESM.docx]

|  | D0 | 4T1 | 4T2 | 4T3 | 4T4 | 7T1 | 7T2 | 7T3 | 7T4 |
| --- | --- | --- | --- | --- | --- | --- | --- | --- | --- |
| Asp | 17.30±2.56 | 19.03±1.33c | 19.87±0.98c | 29.16±0.91b | 37.61±0.49a | 24.32±1.76bc | 20.75±1.84c | 30.03±1.66b | 43.11±3.41a |
| Ser | 14.66±1.57 | 23.79±0.97ab | 24.74±0.90a | 11.95±0.37 | 16.27±0.85c | 23.02±1.07  a | 17.00±0.95bc | 17.30±0.55bc | 18.23±0.88b |
| Glu | 117.42±9.05 | 116.80±4.25c | 144.20±4.21c | 153.77±28.43b | 187.83±8.48a | 152.93±5.22bc | 91.75±4.57 | 162.08±6.70ab | 171.56±8.71a |
| Gly | 6.64±058 | 2.18±0.07a | 1.85±0.09b | 1.39±0.02c | 1.60±0.17bc | 1.27±0.06b | 0.86±0.04 | 1.50±0.04a | 1.13±0.06c |
| Ala | 8.05±0.68 | 16.48±0.63b | 14.13±0.77bc | 14.07±0.31bc | 22.55±1.82a | 15.66±0.53bc | 7.02±0.34 | 15.82±0.63b | 20.12±1.04a |
| Cys | 0.54±0.04 | 0.57±0.04a | 0.32±0.28abc | 0.00c | 0.57±0.21ab | 0.00c | 0.42±0.00c | 0.48±0.01ab | 0.52±0.01a |
| Val | 5.7±0.25 | 4.6±0.11bc | 7.95±0.60a | 4.34±0.15bc | 4.94±0.11b | 8.59±0.28a | 3.45±1.09c | 3.26±0.22c | 5.97±0.39b |
| Met | 0.13±0.01 | 0.00c | 0.08±0.07b | 0.00c | 0.09±0.00a | 0.00 | 0.00 | 0.00 | 0.00 |
| Ile | 2.85±0.30 | 1.08±0.08c | 3.27±0.10a | 1.17±0.03c | 1.61±0.17b | 3.35±0.10a | 0.75±0.04 | 1.31±0.00c | 2.60±0.23b |
| Leu | 1.83±0.45c | 1.06±0.15a | 1.95±0.10c | 1.18±0.05ba | 1.87±0.05 | 1.83±0.08b | 0.86±0.12c | 1.09±0.08bc | 3.00±0.63a |
| Tyr | 2.24±0.01 | 0.00 | 0.00 | 0.00 | 0.00 | 0.00 | 0.00 | 0.00 | 1.64±0.00a |
| Phe | 6.67±0.29 | 8.14±1.37ab | 8.62±0.26a | 5.44±0.22 | 7.84±0.27abc | 8.45±0.09a | 5.01±0.08c | 6.55±1.58abc | 7.96±0.33ab |
| GABA | 0.11±0.01 | 0.51±0.01c | 1.28±0.02b | 0.20±0.02c | 2.58±0.09a | 1.13±0.03b | 0.16±0.01c | 0.33±0.19c | 4.90±0.24a |
| Lys | 4.19±0.41 | 4.53±1.34c | 6.99±0.49a | 4.09±0.08c | 6.76±0.37ab | 4.66±0.11bc | 1.41±0.06 | 6.26±1.51ab | 7.43±0.31a |
| His | 3.29±0.29 | 6.13±0.02a | 5.04±0.13abc | 2.89±0.07 | 6.04±0.31ab | 4.89±0.16ab | 1.89±0.11c | 3.95±1.62abc | 5.71±0.30a |
| Trp | 0.62±0.06 | 1.67±0.20a | 1.03±0.06bc | 0.67±0.02 | 1.04±0.02b | 1.09±0.04ab | 1.01±0.07abc | 0.99±0.25abc | 1.17±0.07a |
| Arg | 57.73±5.35 | 37.12±0.98 | 73.18±12.82ab | 82.73±2.00a | 71.22±5.37abc | 14.98±0.84c | 6.43±0.70c | 108.92±4.48ab | 109.28±6.68a |

Table 5: The dynamic changes of seventeen free amino acids in the tea leaves under the four treatments (T1, T2, T3, T4). (dry weight, mg/100g FW)
