# Supplementary figures and images for: Physiological and iTRAQ-based proteomic analyses reveal the function of exogenous γ-aminobutyric acid (GABA) in improving tea plant (Camellia sinensis L.) tolerance at cold temperature
Source: BMC Plant Biol. 2019 Jan 30;19:43. doi: 10.1186/s12870-019-1646-9 (PMC6354415; doi:10.1186/s12870-019-1646-9)

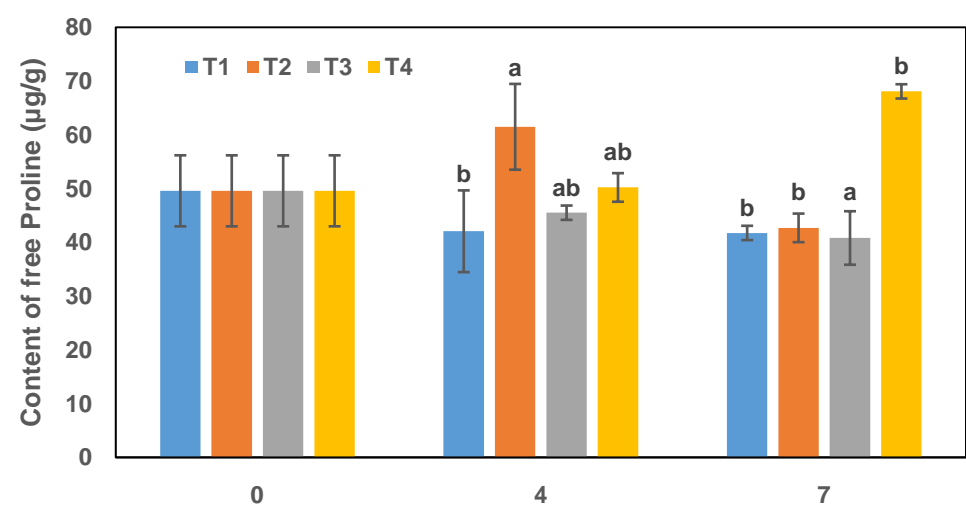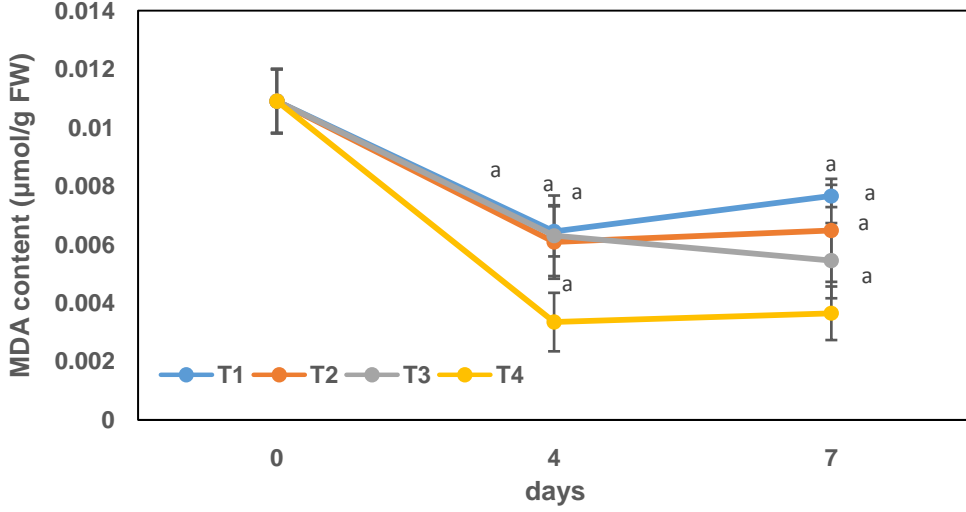

Supplement: Supplementary file 9 — Figure S1: Changes in free proline content and MDA activity during the four treatments (25 °C, 25 °C + GABA, 4 °C and 4 °C + GABA for T1, T2, T3 and T4, respectively). All experiments were performed in triplicate. Data represent the mean value ± standard deviation. Means with different letters are significantly different from each other (p ≤ 0.05). MDA, malonaldehyde. (PDF 153 kb) [file 12870_2019_1646_MOESM9_ESM.pdf]

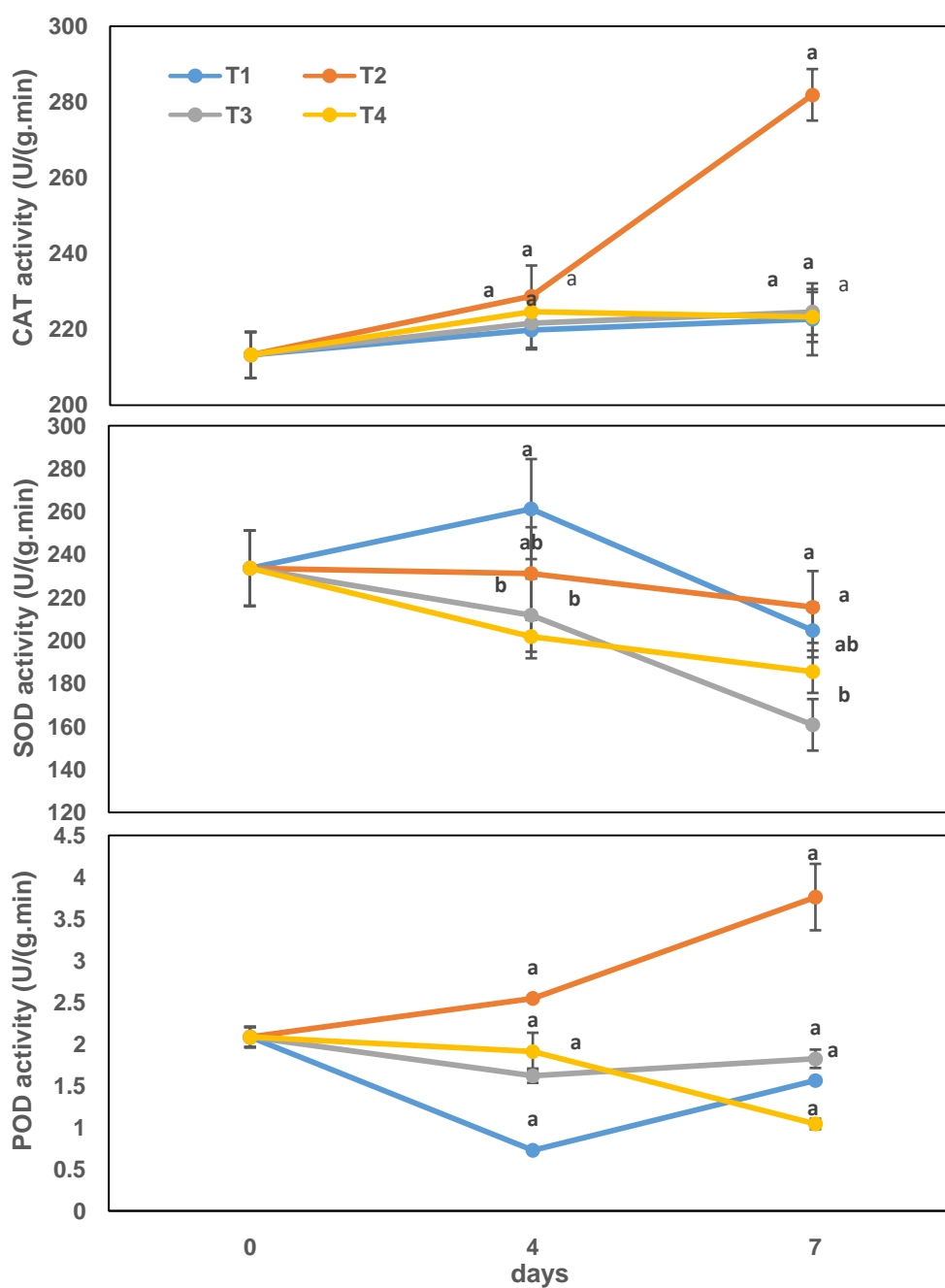

Supplement: Supplementary file 10 — Figure S2: Changes in antioxidant enzyme activity during the four treatments (25 °C, 25 °C + GABA, 4 °C and 4 °C + GABA for T1, T2, T3 and T4, respectively). Data represent the mean value ± standard deviation. Means with different letters are significantly different from each other (p ≤ 0.05). CAT, catalase; SOD, superoxide dismutase; POD, peroxidase. (PDF 167 kb) [file 12870_2019_1646_MOESM10_ESM.pdf]

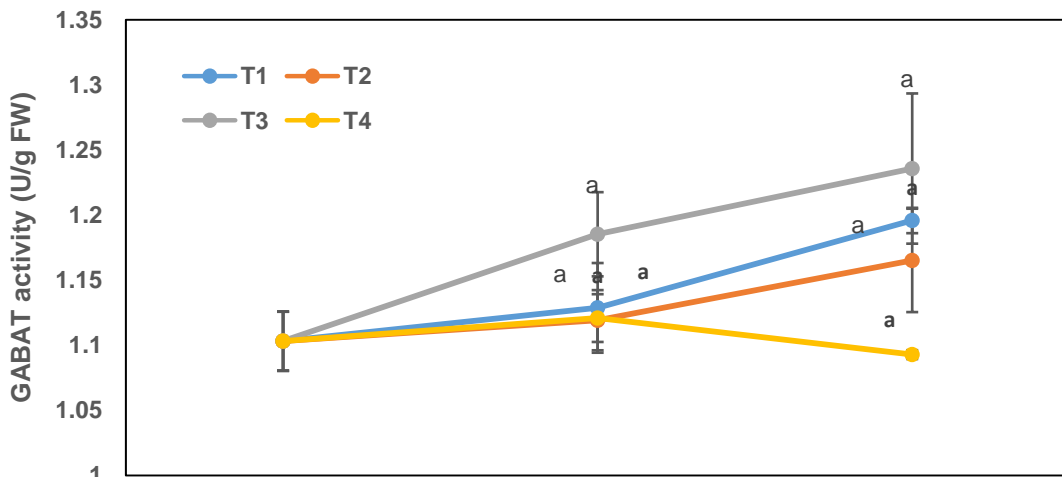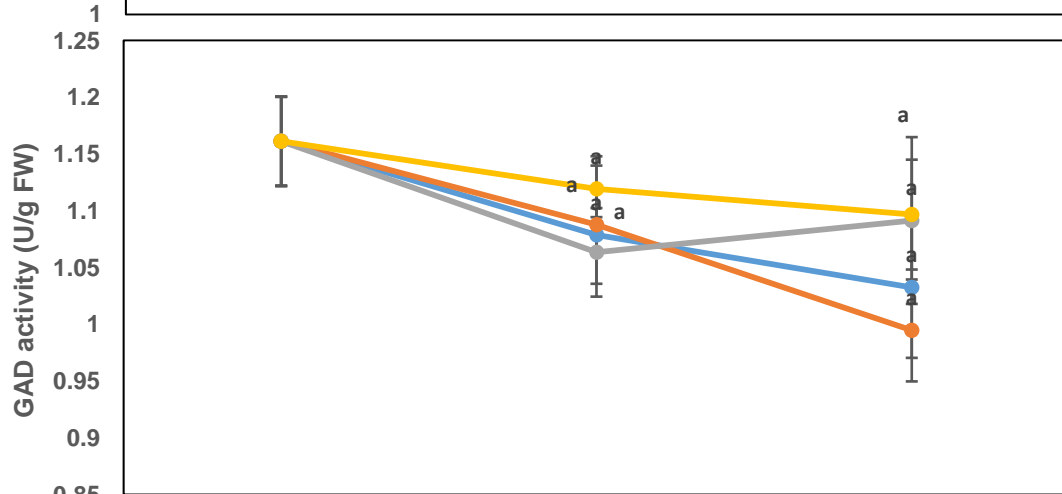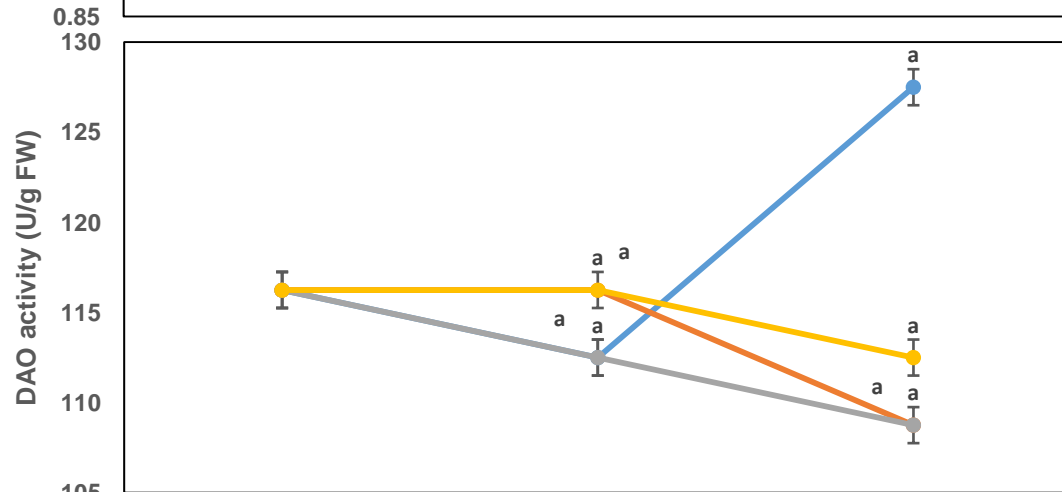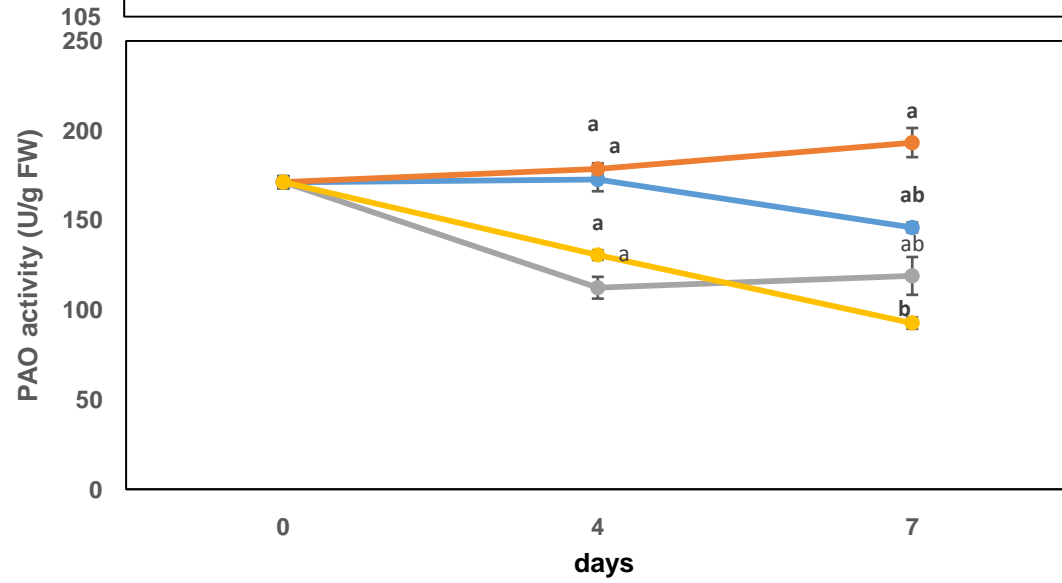

Supplement: Supplementary file 11 — Figure S3: Changes in DAO, PAO, GAD and GABA-T activity. Data represent the mean value ± standard deviation. Means with different letters are significantly different from each other (p ≤ 0.05). DAO, diamine oxidase; PAO, polyamine oxidase; GAD, glutamate decarboxylase; GABA-T, GABA transaminase. (PDF 170 kb) [file 12870_2019_1646_MOESM11_ESM.pdf]

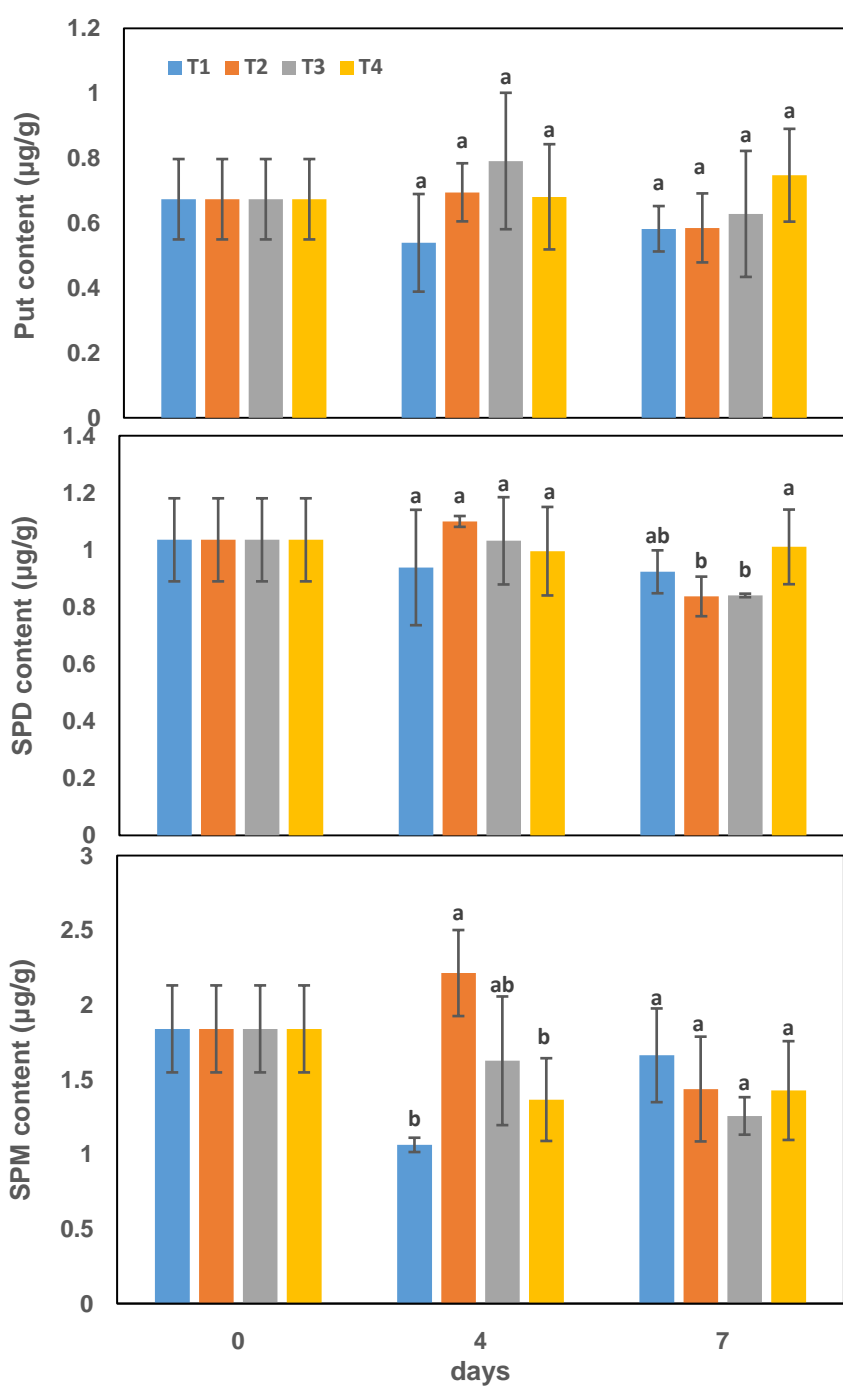

Supplement: Supplementary file 12 — Figure S4: Changes in polyamines contents during the four treatments (25 °C, 25 °C + GABA, 4 °C and 4 °C + GABA for T1, T2, T3 and T4, respectively). Data represent the mean value ± standard deviation. Means with different letters are significantly different from each other (p ≤ 0.05). Put, putrescine; Spd, spermidine; Spm, spermine. (PDF 158 kb) [file 12870_2019_1646_MOESM12_ESM.pdf]
